# Surface chemistry but not aspect ratio mediates the biological toxicity of gold nanorods *in vitro* and *in vivo*

**DOI:** 10.1038/srep11398

**Published:** 2015-06-22

**Authors:** Jiali Wan, Jia-Hong Wang, Ting Liu, Zhixiong Xie, Xue-Feng Yu, Wenhua Li

**Affiliations:** 1 College of Life Sciences, Wuhan University, Wuhan 430072, P R China; 2 Key Laboratory of Artificial Micro- and Nano-Structures of Ministry of Education, School of Physics and Technology, Wuhan University, Wuhan 430072, China; 3 Guangdong Key Laboratory of Nanomedicine, CAS Key Lab of Health Informatics, Institute of Biomedicine and Biotechnology, Shenzhen Institutes of Advanced Technology, Chinese Academy of Sciences, Shenzhen 518055, China

## Abstract

Gold nanorods are a promising nanoscale material in clinical diagnosis and treatment. The physicochemical properties of GNRs, including size, shape and surface features, are crucial factors affecting their cytotoxicity. In this study, we investigated the effects of different aspect ratios and surface modifications on the cytotoxicity and cellular uptake of GNRs in cultured cells and in mice. The results indicated that the surface chemistry but not the aspect ratio of GNRs mediates their biological toxicity. CTAB-GNRs with various aspect ratios had similar abilities to induce cell apoptosis and autophagy by damaging mitochondria and activating intracellular reactive oxygen species (ROS). However, GNRs coated with CTAB/PSS, CTAB/PAH, CTAB/PSS/PAH or CTAB/PAH/PSS displayed low toxicity and did not induce cell death. CTAB/PAH-coated GNRs caused minimally abnormal cell morphology compared with CTAB/PSS and CTAB/PSS/PAH coated GNRs. Moreover, the intravenous injection of CTAB/PAH GNRs enabled the GNRs to reach tumor tissues through blood circulation in animals and remained stable, with a longer half-life compared to the other GNRs. Therefore, our results demonstrated that further coating can prevent cytotoxicity and cell death upon CTAB-coated GNR administration, similar to changing the GNR aspect ratio and CTAB/PAH coated GNRs show superior biological properties with better biocompatibility and minimal cytotoxicity.

In the past few decades, nanomaterials have been extensively studied and have shown many important potential applications in biomedicine. Owing to their good biocompatibility, relative ease of synthesis and special physicochemical properties, gold nanorods (GNRs) have become one of the most promising nanoscale materials for clinical diagnosis and treatment, such as near-infrared imaging (NIR), Raman signal enhancement, tumor photothermal therapy, molecular detection, biosensing, and drug and gene delivery^1^,^2^,^3^,^4^,^5^,^6^,^7^. Compared with conventional spherical nanoparticles, GNRs have shown better properties for biomedical applications, particularly due to their unique optical features^2^,^8^,^9^. The optical properties of GNRs are controlled by their shape and degree of aggregation^10^. GNRs can exhibit variable plasmon bands depending on their aspect ratio (length/width ratio)^11^.

Cetyltrimethylammonium bromide (CTAB) is the most convenient and common surfactant for the synthesis of long gold nanorods. CTAB not only allows further surface modification but also can be used as a surfactant stabilizer for synthesis and deposition. Seed-mediated methods using CTAB result in tightly packed CTAB bilayers on the surfaces of the gold nanorods; this positively charged layer creates mutual repulsions and prevents aggregation, keeping the nanorods in a stable form. However, CTAB is a highly toxic cationic surfactant^12^,^13^. Synthetic GNRs with CTAB conjugates are toxic to cultured cells and to animals^14^,^15^,^16^. Although many reports have shown that the toxicity of colloidal GNRs depends on the particle concentration, size, shape, surface modification and even coating methods and medium^16^,^17^,^18^,^19^,^20^, studies examining the *in vitro* and *in vivo* toxicity of GNRs are rare and controversial. A comparison of the toxicity of gold nanorods of various sizes and with different coatings would help to identify target sites that may be at risk from nanoparticle exposure.

The common biological method of evaluating toxicity is to measure cytotoxicity *in vitro* and systemic toxicity *in vivo*. Apoptosis (type I programmed cell death) and autophagy (type II programmed cell death) are the two most important forms of programmed cell death. Many chemotherapy drugs or other agents can effectively induce cell apoptosis or autophagy^21^,^22^,^23^,^24^, thus showing cytotoxicity. However, non-specific cytotoxicity, for example, toxicity against functioning, normal cells, will result in systemic side effects. The issues related to the potential toxicity of gold nanorods were studied in the early days of nanotechnology development. However, the potential toxicity and toxicity mechanisms of many types of gold nanorods is not fully understood.

In this study, we synthesized gold nanorods with different aspect ratios and surface modifications and investigated the influences of these physicochemical properties on cytotoxicity and cellular uptake in cultured cells and in mice. The results indicated that CTAB-coated GNRs, regardless of their aspect ratio, are relatively toxic, inducing cell apoptosis and autophagy. Damage to mitochondria and the activation of intracellular reactive oxygen species (ROS) play roles in inducing cell apoptosis and autophagy. However, further modification of the surfaces of CTAB-coated GNRs with polystyrene sulfonate (PSS), poly allylamine hydrochloride (PAH), or both (PSS/PAH) dramatically decreased their toxicity. *In vivo* studies showed that GNRs coated with CTAB/PAH were less toxic to mice, and these GNRs could be distributed to the liver, spleen, lungs and other tissues. Therefore, our results demonstrate that further coating could prevent mitochondria-mediated cell apoptosis and autophagy in response to CTAB-coated GNRs. Thus, surface chemistry and not aspect ratio mediates the biological toxicity of GNRs *in vitro* and *in vivo*.

## Results

### Synthesis and characterization of GNRs

**Figure 1:**
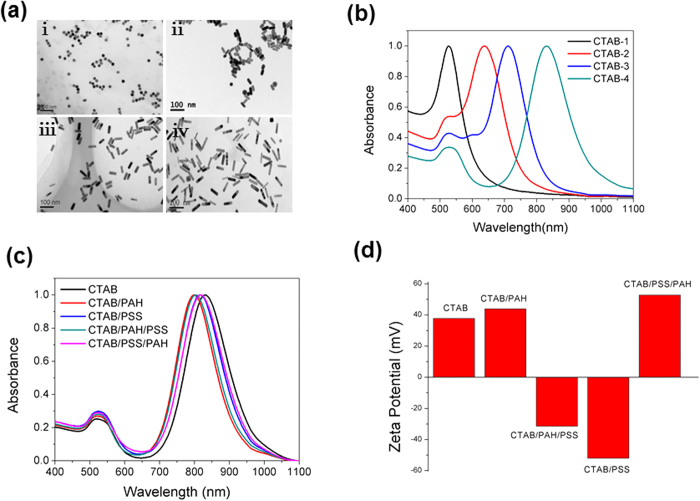
Characterization of GNRs by transmission electron microscopy (TEM) and visible and near-infrared absorption spectra. (a) TEM images of CTAB-coated GNRs with different aspect ratios: (i) CTAB-1, (ii) CTAB-2, (iii) CTAB-3, and (iv) CTAB-4. (**b**) Representative UV-Vis-NIR absorption spectra of CTAB-coated GNRs with four different aspect ratios. (**c**) UV-Vis-NIR absorption spectra of GNRs with CTAB, CTAB/PSS, CTAB/PAH, CTAB/PSS/PAH and CTAB/PAH/PSS coatings. (**d**) Zeta potentials of the GNRs with different coatings.

GNRs with different aspect ratios and different surface modifications were synthesized and characterized as described in the Materials and Methods section. The morphologies of CTAB-coated GNRs with four different aspect ratios were observed by TEM and designated as CTAB-1, CTAB-2, CTAB-3, and CTAB-4 according to increasing aspect ratio (Fig. 1a). Representative UV-Vis-NIR absorption spectra are shown in Fig. 1b. To examine the biocompatibility of the different GNR coatings, we synthesized polyelectrolyte-coated GNRs functionalized with CTAB/PSS, CTAB/PAH, CTAB/PSS/PAH and CTAB/PAH/PSS, which all displayed similar UV-Vis-NIR absorption spectra (Fig. 1c). The zeta potentials of these different GNRs showed that the CTAB-coated, CTAB/PAH-coated and CTAB/PSS/PAH coated GNRs were positively charged, while the CTAB/PSS-coated and CTAB/PAH/PSS-coated GNRs were negatively charged (Fig. 1d).

PSS and PAH are typical polyelectrolytes to form layers onto the CTAB-GNRs surface. The interactions between them and CTAB-GNRs have been well studied previously^25^,^26^,^27^. On the one hand, the polyanion PSS can electrostatically associate with the CTAB-coated GNRs. On the other hand, it has been reported that amines bind to gold nanoparticles through the amine functionality via a weak covalent bond^28^. Since PAH contains uncharged amine groups (-NH2), it can bind onto the CTAB-GNRs through the amine functionalities, despite having the same charge as the nanoparticles^25^,^26^,^27^.

### Cytotoxicity of CTAB-GNRs on tumor cells and normal cells

**Figure 2:**
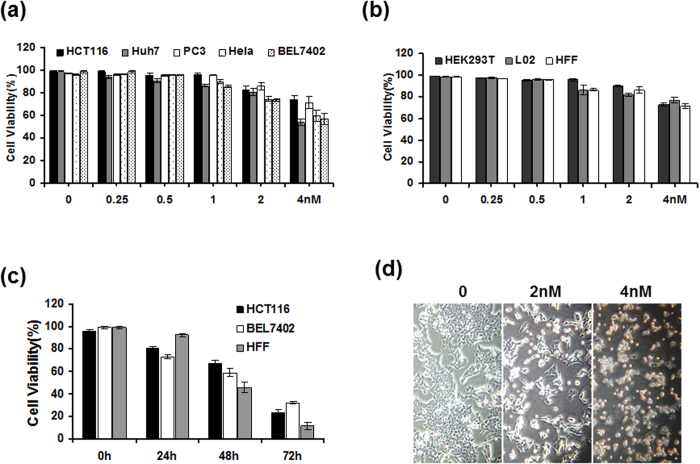
Cell viability induced by CTAB-coated GNRs. The data represent the average of at least three independent experiments ± SD. (**a**) Cancer cell lines (HCT116, Huh7, PC3, Hela, BEL7402) and (**b**) immortalized nonmalignant cell lines (HEK293T, L02, HFF) were treated with increasing concentrations of CTAB-coated GNRs for 24 h. Cell viability was determined by trypan blue exclusion assays. (**c**) Time-course analysis of cell viability following the treatment of HCT116, BEL7402 and HFF cells with 2 nM CTAB-coated GNRs. (**d**) Phase microscopy of HCT116 cells treated with CTAB-coated GNRs for 24 h at 2 and 4 nM, respectively.

To determine whether the CTAB-GNRs were toxic to cells, different types of tumor cells (HCT116, Huh7, PC3, Hela and BEL7402) and non-malignant transformed cells (HEK293T, L02 and HFF) were exposed to various concentrations of CTAB-GNRs (0.25, 0.5, 1, 2, 4 nM) for 24 hours and to 2 nM CTAB-GNRs for different times (24 h, 48 h, 72 h). Cell viability was determined by a trypan blue dye exclusion assay. The concentration- and time-dependent cytotoxicity of the particles could be observed (Fig. 2a–c), where maximal effects were found after 72 h of incubation at the highest CTAB-GNR concentration. Moreover, the cytotoxicity of the CTAB-GNRs toward tumor cells was higher than that toward non-malignant cells. Consistent with the cell viability results, phase microscopic analysis also showed that the CTAB-GNR-treated cells exhibited features of death (Fig. 2d). Therefore, the cell viability results indicated that the CTAB-GNRs are toxic to cultured cells.

### CTAB-GNRs induce apoptosis in human tumor cells

**Figure 3:**
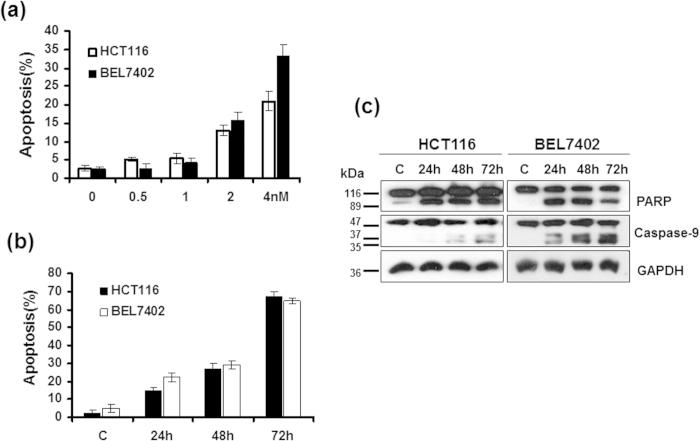
Treatment with CATB-coated GNRs results in apoptosis in cells. (**a**) Dose-dependent cell apoptosis after treatment with CTAB-coated GNRs for 24 h. (**b**) Time dependence of cell apoptosis after treatment with CTAB-coated GNRs at 2 nM. (**c**) Western blot analysis to detect apoptosis-related PARP and caspase-9 proteins. GAPDH was measured as a loading control. Cropped lines are displayed in the figure and full-length blots are presented in Supplementary Figure 1. The gels have been run under the same experimental conditions.

Apoptosis is an important form of cell death and is the most common mechanism for targeted chemotherapies that induce cancer cell death^29^. To determine whether the CTAB-GNRs could trigger apoptosis, HCT116 and BEL7402 cells were treated with different concentrations of CTAB-GNRs (0.5, 1, 2, 4 nM) for 24 hours and with 2 nM CTAB-GNRs for different times (24, 48, 72 h). Apoptosis was evaluated by a sub-G1 assay after staining with propidium iodide (PI). The results indicated that the CTAB-GNRs induced dose-dependent apoptosis (Fig. 3a). Moreover, with increasing incubation time, the cytotoxicity of the CTAB-GNRs also increased (Fig. 3b). In agreement with the FACS analysis, a Western blot also indicated that the CTAB-GNRs activated the apoptotic proteins PARP and caspase-9 (Fig. 3c). These data suggested that the CTAB-GNRs’ cytotoxicity could be mediated through the induction of apoptosis.

### CTAB-GNRs induce Akt-independent autophagy in human cells

**Figure 4:**
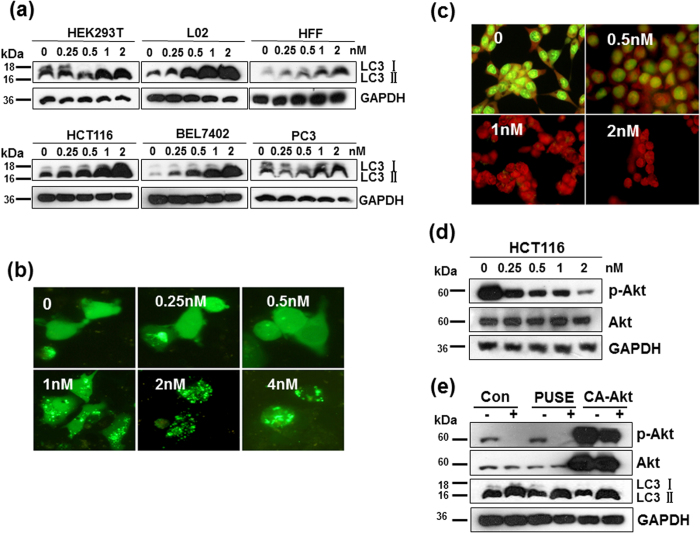
CTAB-coated GNRs induced autophagy in cells. Cells were treated with 0.25–2 nM CTAB-coated GNRs for 24 h. The autophagy of HCT116 cells induced by CTAB-coated GNRs was Akt independent. (**a**) Western blot analysis of LC3 protein levels in cancer cell lines as well as in immortalized nonmalignant cell lines. Cropping lines are used in the figure. Full-length blots are presented in Supplementary Figure 2. The gels have been run under the same experimental conditions. (**b**) Representative GFP-LC3 fluorescent punctate dot images in HCT116. (**c**) Detection of acidic vesicular organelles with acridine orange staining. (**d**) Western blot analysis of Akt in cells treated with 0.25–2 nM CTAB-coated GNRs for 24 h. Cropping lines are used in the figure. Full-length blots are presented in Supplementary Figure 2. The gels have been run under the same experimental conditions. (**e**) Cells transfected with vehicle plasmid (pUSE) or constitutively active Akt (CA-Akt) were incubated with or without 2 nM CTAB-coated GNRs for 24 h and then analyzed for LC3 and Akt by Western blot. Cropping lines are used in the figure. Full-length blots are presented in Supplementary Figure 2. The gels have been run under the same experimental conditions.

Autophagy is a cellular process involved in protein and organelle degradation and in the recycling of cellular components to ensure survival during starvation and other stresses^30^,^31^. To investigate whether the CTAB-GNRs could induce autophagy in human non-malignant transformed cells (HEK293T, L02, HFF) or cancer cells (HCT116, BEL7402, PC3), we first examined the protein level of LC3-II, a cleaved form of LC3 that acts as an established molecular indicator of autophagy. As shown in Fig. 4a, Western blot analysis showed that 1 nM CTAB-GNR treatment significantly increased the LC3-II protein level in all detected cells. To further confirm the induction of cell autophagy by the CTAB-GNRs, HCT116 cells were treated with CTAB-GNRs for 24 h after being transfected with the EGFP-LC3 plasmid. Microscopic examination showed the characteristic punctate fluorescent pattern of LC3-GFP (Fig. 4b), suggesting the formation of autophagosomes and the occurrence of autophagy. Moreover, HCT116 cells stained with acridine orange after being treated with CTAB-GNRs showed considerable red fluorescence, suggesting the formation of numerous acidic autophagolysosome vacuoles (AVOs) (Fig. 4c). The above data demonstrated that the CTAB-GNRs could trigger autophagy in HCT116 cells.

Akt is a critical kinase that regulates downstream of mTOR and is involved in a variety of biological processes, including cell apoptosis and autophagy through transduction cascades^32^,^33^. Next, we investigated whether the autophagy of HCT116 cells induced by the CTAB-GNRs was mediated through Akt. As shown in Fig. 4d and e, although treatment with CTAB-GNRs decreased Akt activation, the overexpression of CA Akt did not rescue the GNR-induced autophagy. Therefore, these results indicate that Akt kinase was not involved in the CTAB-GNR-induced autophagy.

### Mitochondria are involved in CTAB-GNR-induced autophagy

**Figure 5:**
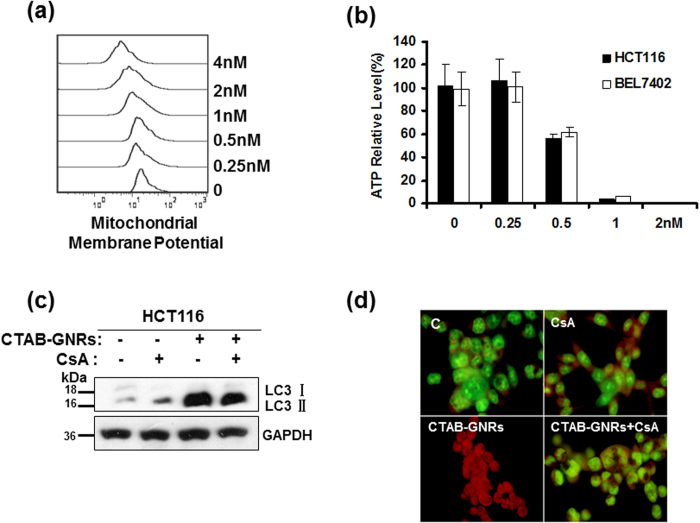
The autophagy induced by CTAB-coated GNRs is mediated through a mitochondrial pathway. (**a**) HCT116 cells were treated with 0.25–4 nM CTAB-coated GNRs for 24 h, and the cells were then subjected to flow cytometry analysis of the mitochondrial membrane potential. (**b**) ATP relative level detected by a luminometer. The ATP level of untreated cells was set at 100%. (**c**) Cells were treated with 2 nM CTAB-coated GNRs alone or in combination with 2 mM CsA for 24 h. The expression of LC3 was detected by Western blotting. Cropping lines are used in the figure. Full-length blots are presented in Supplementary Figure 3. The gels have been run under the same experimental conditions. (**d**) Detection of acidic vesicular organelles with acridine orange staining.

Mitochondrial homeostasis is critical in regulating cell physiology as well as cell survival, including during cell apoptosis and autophagy^34^,^35^. Next, we examined whether mitochondrial events were associated with the CTAB-GNR-induced autophagy. We detected changes in the mitochondrial membrane potential (∆ψm), which resulted from mitochondrial membrane depolarization and led to an increase in the permeability of the outer membrane. As shown in Fig. 5a, a ∆ψm decrease was observed in the HCT116 cells following CTAB-GNR treatment. A similar result was also obtained for the ATP level (Fig. 5b). However, cyclosporine A (CsA), a mitochondrial membrane potential stabilizer, at least partially abrogated CTAB-GNR-induced autophagy (Fig. 5c,d). These results therefore suggest that mitochondria are involved in CTAB-GNR-induced autophagy.

### The autophagy induced by CTAB-GNRs is mediated through intracellular reactive oxygen species (ROS)

**Figure 6:**
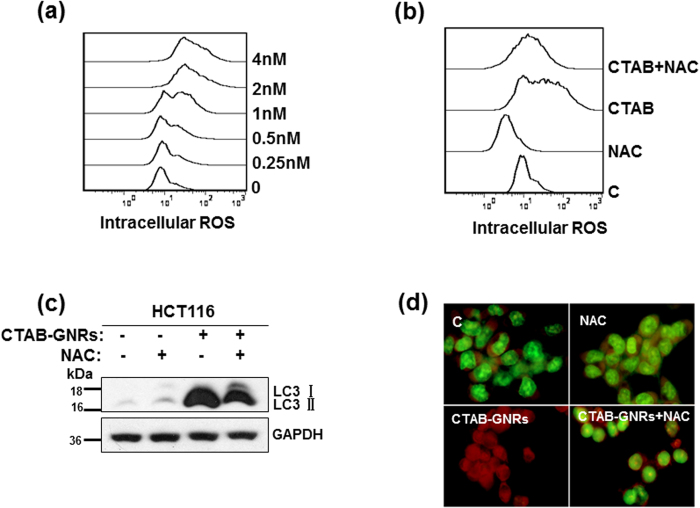
Intracellular reactive oxygen species (ROS) are involved in the autophagy induced by CTAB-coated GNRs. (**a**) HCT116 cells were treated with 0.25–4 nM CTAB-coated GNRs for 24 h, and the cells were then subjected to flow cytometry analysis of intracellular ROS. (**b**) Cells were treated with 2 nM CTAB-coated GNRs alone or in combination with 10 mM NAC for 24 h. Intracellular ROS levels were detected by flow cytometry. (**c**) Western blot analysis of the LC3 protein level. Cropping lines are used in the figure. Full-length blots are presented in Supplementary Figure 4. The gels have been run under the same experimental conditions. (**d**) Detection of acidic vesicular organelles with acridine orange staining.

ROS, which are mainly produced in the mitochondria, take part in the regulation of physiological cell signaling. Many studies have shown that ROS can induce cellular apoptosis and/or autophagy if produced excessively in certain types of cancer cells^36^,^37^,^38^. Thus, we next determined whether CTAB-GNR-induced autophagy is related to ROS activation in HCT116 cells. We measured the intracellular ROS generated by treatment of the HCT116 cells with various concentrations of CTAB-GNRs. As shown in Fig. 6a, dose-dependent ROS accumulation was observed. However, the free radical scavenger NAC markedly abrogated CTAB-GNR-induced ROS generation, which further confirms that the CTAB-GNRs caused intracellular ROS accumulation (Fig. 6b). Moreover, pretreatment with NAC for 1 h significantly decreased the LC3-II protein level and abolished the formation of acidic autophagolysosome vacuoles induced by the CTAB-GNRs (Fig. 6c,d), which suggested that NAC could rescue the cells from CTAB-GNR-induced autophagy. These data demonstrated that the autophagy induced by the CTAB-GNRs is mediated through the production of intracellular reactive oxygen species.

### The cytotoxicity of CTAB-GNRs is independent of their aspect ratio

**Figure 7:**
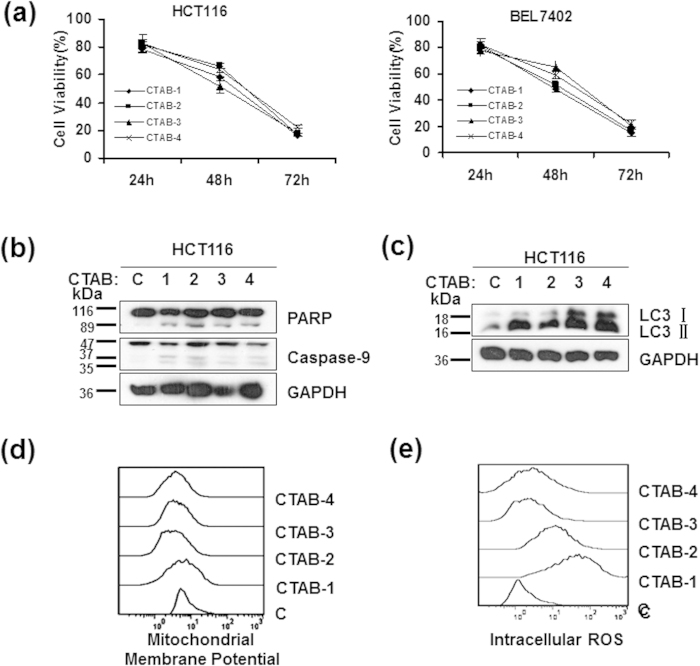
Cytotoxicity studies of cells treated with GNRs of different aspect ratios. Cells were treated with GNRs with four different ARs coated with CTAB (CTAB-1, CTAB-2, CTAB-3, CTAB-4) at 2 nM for 24 h, 48 h or 72 h. (**a**) Cell viability was determined by a trypan blue exclusion assay after 24, 48, or 72 h of treatment with CTAB-coated GNRs with different aspect ratios.Western blot analysis of the cell apoptosis marker PARP, caspase-9 (**b**) and the autophagy marker LC3 (**c**). Cropping lines are used in the figure. Full-length blots are presented in Supplementary Figure 5. The gels have been run under the same experimental conditions.Flow cytometry analysis of (**d**) intracellular ROS and (**e**) mitochondrial membrane potential.

It has been reported that the cellular uptake of gold nanorods is highly dependent on their aspect ratio^17^,^39^. To determine whether the cytotoxicity of CTAB-GNRs is associated with their aspect ratio, HCT116 cells were treated with different aspect ratio CTAB-GNRs (aspect ratios of 1, 2, 3, and 4) for 24, 48 and 72 hours. The data showed that the CTAB-GNRs had similar abilities to induce cell death regardless of aspect ratio (Fig. 7a). Moreover, Western blot analysis showed that the CTAB-GNRs of different aspect ratios could induce the cleavage of caspase-9 and PARP and increase the LC3-II levels, which indicated that the cells had undergone apoptosis and autophagy (Fig. 7b,c). Consistent with the cell viability and Western blot results, the CTAB-GNRs with different aspect ratios had similar abilities to decrease the mitochondrial membrane potential (Fig. 7d), although they exhibited different abilities to activate intracellular ROS (Fig. 7e). These results indicated that the aspect ratio of CTAB-GNRs has little effect on their cytotoxicity.

### Surface chemistry is the key factor determining the cytotoxicity of gold nanorods

**Figure 8:**
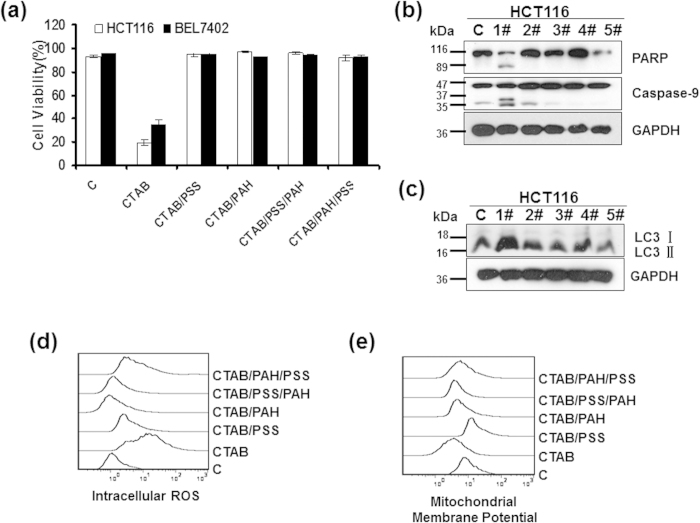
Cytotoxicity studies of cells treated with GNRs with different coatings. Cells were treated with GNRs with different coatings (1# = CTAB, 2# = CTAB/PSS, 3# = CATB/PAH, 4# = CTAB/PSS/PAH, 5# = CTAB/PAH/PSS) at 2 nM for 24 h (**b,d,e**) or 72 h (**a,c**). (**a**) Cell viability was determined by trypan blue exclusion assays. Western blot analysis of the cell apoptosis marker PARP, caspase-9 (**b**) and the autophagy marker LC3 (**c**). Cropping lines are used in the figure. Full-length blots are presented in Supplementary Figure 6. The gels have been run under the same experimental conditions. Flow cytometry analysis of (**d**) intracellular ROS and (**e**) the mitochondrial membrane potential.

Surface modifications have been extensively used to improve the biocompatibility and stability of gold nanorods. The cellular uptake of GNRs synthesized by the seed-mediated method is dramatically influenced by their surface properties^16^,^17^. Thus, we investigated the influence of the GNR surface coatings on their toxicity. As shown in Fig. 8a, compared with the single CTAB coatings, the multi-coated GNRs exhibited reduced cytotoxicity. The GNRs coated with CTAB/PSS, CTAB/PAH, CTAB/PSS/PAH or CTAB/PAH/PSS induced almost no cell death. Similarly, the PARP, caspase-9 and LC3 levels indicated that the GNRs did not induce apoptosis or autophagy in the HCT116 cells (Fig. 8b,c). In addition, we also examined the effects of these multi-coated GNRs on the mitochondrial membrane potential and intracellular ROS levels (Fig. 8d,e). Consistent with the cell survival studies, the GNRs with multiple coatings (CTAB/PSS, CTAB/PAH, CTAB/PSS/PAH or CTAB/PAH/PSS) had almost no effect on the functioning of the mitochondria. These data suggest that multi-coated GNRs are less toxic than are CTAB-GNRs. Therefore, the surface modification of GNRs is the critical factor determining their cytotoxicity.

### TEM images and intracellular localization of GNRs

**Figure 9:**
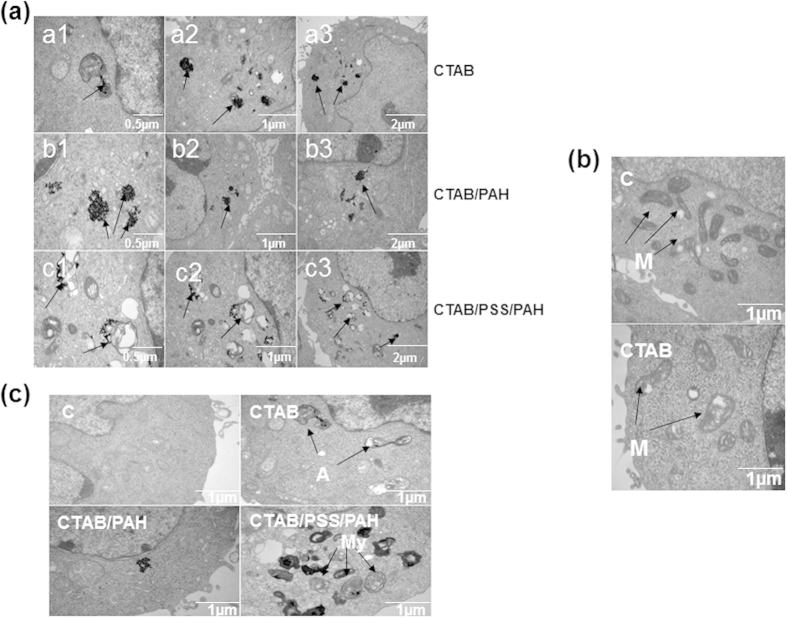
TEM images of GNRs and their intracellular localization. Cells were incubated with 2 nM GNRs for 24 h (**a–c**). (**a**) The GNRs formed distinct dark aggregates (black arrows) in cells and cell organelles. (**b**) CTAB-coated GNRs caused the swelling of mitochondria. (**c**) Autophagosomes (black arrows) were observed by TEM in cells treated with CTAB-coated GNRs. A is short for autophagosomes.

Although we found that the CTAB-GNRs could trigger cell apoptosis and autophagy, it was still unclear whether these GNRs could enter the cells. We used three different coated GNRs at a 2 nM concentration to treat HCT116 cells for 24 hours, and then observed the cell morphology and status of the GNRs. As shown in Fig. 9a, the three types of GNRs (CTAB, CTAB/PAH, CTAB/PSS/PAH) were able to fully enter into the cells and localize to the cytoplasm, lysosome and endosome. Treatment with the CTAB-GNRs caused mitochondrial swelling and the production of autophagosomes; however, no mitochondrial swelling or autophagosomes appeared following treatment with CTAB/PAH or CTAB/PSS/PAH coated GNRs, consistent with the above autophagy results (Figs 9b,c and 4). Moreover, we found that treatment with CTAB/PSS/PAH coated GNRs caused the cells to produce large amounts of myelin bodies. TEM observations showed that the CTAB/PAH coated GNRs caused minimal abnormal cell morphology, suggesting that this bilayer coating provides better biocompatibility and lower cytotoxicity.

### Biotoxicity and biodistribution of CTAB/PAH-coated GNRs* in vivo*

**Figure 10:**
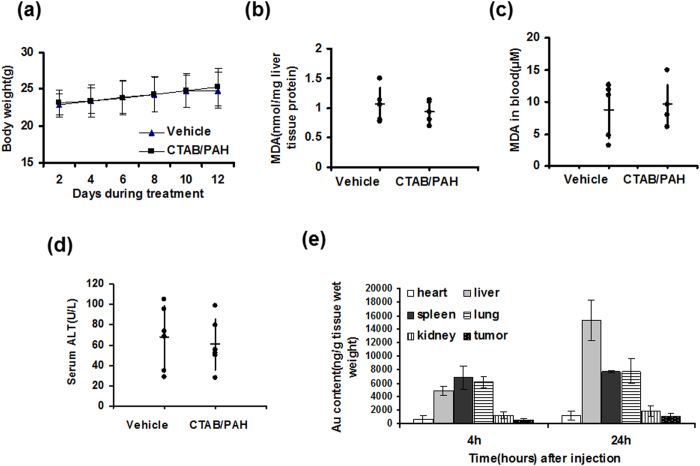
Biotoxicity and biodistribution of CTAB/PAH-coated GNRs *in vivo*. Twelve 6-week-old male BALB/c mice were used to study the toxicity of CTAB/PAH-coated GNRs (**a–c**). (**a**) The body weights were measured every 2 days. MDA assay of (**b**) liver tissues and (**c**) blood samples treated with vehicle or with CTAB/PAH-coated GNRs in mice. (**d**) Blood samples from the mice were subjected to serum ALT activity analysis. (**e**) Biodistribution of CTAB/PAH-coated GNRs in mice for 4 h and 24 h after intravenous injection. Inductively coupled plasma mass spectrometry (ICP-MS) was employed to measure the total amount of gold in the heart, liver, spleen, lungs, kidneys and tumor tissue. The data are described as the mean values and standard deviations (n = 3).

Based on the above results, we concluded that the CTAB/PAH-coated GNRs had low toxicity and did not induce cell death or mitochondrial damage. Next, we examined the biotoxicity and biodistribution of the CTAB/PAH-coated GNRs *in vivo*. Twelve male 6-week-old BALB/c mice were used to study the toxicity of the GNRs by measuring the body weight, MDA level and ALT activity. The mice were injected intravenously with 200 μL of a 0.9% saline solution containing GNRs at a dose of 560 μg/kg weight every other day for two weeks. Liver tissues and blood samples from the mice were harvested after all mice were sacrificed. The results showed that the CTAB/PAH-coated GNRs did not cause weight loss in mice, did not stimulate the level of reactive oxygen species in liver tissues or blood samples, and did not increase the serum ALT activity (Fig. 10a–d). These data suggested that the CTAB/PAH-coated GNRs had low toxicity *in vivo*. To examine whether the GNRs reached the tumor tissues through blood circulation, we used eight subcutaneous tumor-bearing mice randomly divided into two groups. We performed the intravenous delivery of 200 μl of a 0.9% saline solution containing CTAB/PAH-coated GNRs at a dose of 560 μg/kg weight. The mice were anesthetized with ether, weighed, and killed at 4 h and 24 h. The heart, liver, spleen, lung, kidney, and tumor tissues were collected, and the biodistribution of the CTAB/PAH-coated GNRs was measured by ICP-MS. As shown in Fig. 10e, at longer treatment times, the content of GNRs in the mice tissues increased. The data indicated that the GNRs were mainly distributed in the liver, spleen and lungs. Additionally, the tumor tissues contained some GNRs. These results reveal that the CTAB/PAH-coated GNRs are stable *in vivo* and have a long half-life (Fig. 10e).

## Discussion

Compared with spherical nanoparticles, gold nanorods, which strongly absorb near-infrared (NIR) light, are more sensitive to changes in the local environment and offer stronger scattering and absorption efficiency per unit volume^8^,^9^. Owing to their special physicochemical properties and biocompatibility, GNRs have been extensively investigated for their potential applications in the fields of biology and medicine^40^,^41^. Currently, GNRs have become a model system for the development of nanoprobes. The most conventional synthesis method for GNRs is the seed-mediated method using CTAB, which is a toxic cationic surfactant^12^,^13^. It is therefore necessary to examine the toxicity mechanism of GNRs and to develop safer GNRs. The particle size, shape, aspect ratio and surface charge and modification are considered to be the most important factors influencing the cytotoxicity of GNRs. Smaller sized particles with higher surface-area-to volume ratios are often considered more dangerous^42^.

The toxicity of a material mainly lies in its potential to induce cell death or cause a systemic pathological response *in vivo*. Apoptosis and autophagy are the two major ways for programmed cell death. Apoptosis has significant roles in maintaining organism homeostasis and metabolic balance and in organ development. Additionally, apoptosis is the most common mechanism for targeted chemotherapies^43^. Autophagy is a basic catabolic mechanism that involves the cell degradation of unnecessary or dysfunctional cellular components, including some proteins and organelles, during starvation or external or internal stimuli^44^. A considerable number of toxic substances can cause cellular stress, including ER stress and ROS, which may lead to the activation of autophagy. In the present study, we examined the toxicity of GNRs by detecting their potential to induce cell apoptosis and autophagy. The results indicated that surface modification is the key factor determining the cytotoxicity of GNRs, while the aspect ratio does not influence their ability to induce cell death. CTAB-GNRs with different aspect ratios (1, 2, 3, 4) had similar abilities to induce HCT116 cell death and destroy mitochondria (Fig. 7). In terms of the surface modification, compared with the GNRs coated with CTAB alone, the GNRs coated with CTAB/PSS, CTAB/PAH, CTAB/PSS/PAH or CTAB/PAH/PSS showed minimal toxicity and had almost no effect on cell viability or mitochondrial damage (Fig. 8). Our data also showed that the primary mechanism of toxicity of the CTAB-GNRs was through the activation of intracellular ROS and mitochondrial damage, which firmly affect cell survival. High-aspect-ratio GNRs are of particular interest for biomedical applications because of their increased scattering contrast^10^. The present study indicated that no obvious difference in toxicity was found among GNRs with different aspect ratios, which suggests that high-aspect-ratio GNRs may be superior in potential applications.

The toxicity of GNRs is also influenced by the surface modification because the surface coating can influence cellular uptake^16^,^17^,^45^,^46^. Some researchers have proposed that nanoparticle internalization occurs via receptor-mediated endocytosis arising from the cellular recognition of proteins in the media^47^,^48^,^49^,^50^,^51^. When cell tries to shield itself from toxicity induced by the rod, different plasma proteins will adsorb on nanoparticles surface. The nanoparticles subsequently enter the cells after adopting the physiochemical properties of the adsorbed protein shell, but it not always worked well. Not all kinds of nanoparticles covered with various surface modifications will enter the cells effectively and safely. Only conjugated with proper biochemical molecules can nanoparticles recognize the proteins on cell membrane. And protein adsorption to the nanoparticles surface can mediate the receptor-mediated endocytosis or the direct penetration of nanomaterial. The potential toxicity and health impact of nanomaterials are essential before these nanomaterials can be used in real clinical settings. Since cytotoxicity was influenced by the physicochemical properties of nanorods, it is very important to utilize surface modifications to control the biological effects of nanoparticles to satisfy safe applications in nanomedicine. GNRs were rapidly coated by proteins and then transported by vesicles into the lysosome^52^. Smaller GNRs are often considered more toxic because they are more likely to be taken up into intracellular locations, such as the nucleus and mitochondria, that cannot be reached by larger materials^42^. In this study, we investigated the internalization of GNRs by transmission electron microscopy and found that three different coated GNRs (CTAB, CTAB/PAH, CTAB/PSS/PAH) can be taken up by HCT116 cells after treatment for 24 hours (Fig. 9). The GNRs were mainly distributed in the cytoplasm, lysosomes and endosomes in the form of aggregates. Considering the cellar uptake of the GNRs and the cell morphology, the CTAB/PAH bilayer-coated GNRs were more biocompatible and showed minimal cytotoxicity. In addition to surface modification, some reports have shown that the cytotoxicity of GNRs is also closely related to the original physicochemical properties of the nanomaterials, for example, the charge and dimensions^53^,^54^. Although here we did not examine the effect of aspect ratio on GNR internalization, previous studies have shown that PDDAC-coated GNRs with an aspect ratio of 4 possesses negligible toxicity and high cellular uptake efficiency^17^.

In addition to performing *in vitro* cell investigations, the absorption, biodistribution, clearance and toxicity of the CTAB/PAH-coated GNRs *in vivo* was evaluated using subcutaneous tumor-bearing mice. GNRs administered via intravenous injection can reach multiple organs and tumor tissues in animals, even when the tumor is relatively small. The distribution in the tissues and organs after 24 hours was greater than that after 4 hours, indicating that the CTAB/PAH-coated GNRs were stable *in vivo* and had a long half-life (Fig. 10). Therefore, CTAB/PAH-coated GNRs are a promising biomedical nanomaterial for photothermal therapy, bioimaging, sensing, drug delivery, and cancer treatment.

## Materials and Methods

### Ethics statement

The present study was approved by Wuhan University. And, the methods were carried out in accordance with the approved guidelines. In addition, all experimental protocol was approved by Wuhan University and written informed consent was obtained from every subject.

### Chemicals and cell culture

Chloroauric acid (HAuCl4•4H2O, 99.99%), silver nitrate (AgNO3, 99.8%), L-ascorbic acid (99.7%), sodium chloride (NaCl, 96.0%), and hydrochloric acid (HCl, 36–38%) were purchased from Sinopharm Chemical Reagent Co. Ltd. (Shanghai, China). Sodium borohydride (NaBH4, 96%), poly(allylamine hydrochloride) (PAH, MW ~15,000 g/mol), and poly(sodium 4-styrenesulfonate) (PSS, MW ~70,000 g/mol) were obtained from Aldrich (USA). Hexadecyltrimethylammonium bromide (CTAB, 99.0%) was purchased from Amresco Inc. (USA). All the chemicals were used as received without further purification. Ultrapure water with a resistivity of approximately 18.25 MΩ•cm was used as the solvent in all experiments.

DCFH-DA (2′, 7′-dichlorofluorescein diacetate) was obtained from Invitrogen (Carlsbad, CA). N-acetyl-L-cysteine (NAC) was purchased from Sigma (St. Louis, MO). Rhodamine 123 (Rh123), cyclosporin A (CsA), acridine orange, GAPDH antibody and HRP-conjugated secondary antibodies (goat anti-rabbit and goat anti-mouse) were purchased from Beyotime (Nantong, China).The antibody against microtubule-associated protein 1 light chain 3 (LC3) was purchased from Sigma, and antibodies against caspase-9, PARP, total-Akt, phospho-Akt (Ser473), total MEK, phospho-MEK, total-ERK, phospho-ERK (Thr202/Tyr204), total-p38, phospho-p38 (Thr180/Tyr182), mTOR, phospho-p70s6k, and phospho-rps6 were purchased from Cell Signaling Technology (Beverly, MA).

Human colon cancer cells (HCT116) were cultured in McCoy’s 5A medium. Human tumor cell lines (BEL7402, Huh7, PC3 and Hela) and immortalized nonmalignant cell lines (L02, HEK293T and HFF) were cultured in Dulbecco’s modified Eagle’s medium (DMEM). All the cells were cultured in culture media supplemented with 10% fetal bovine serum (FBS, Hyclone), penicillin (100 units/mL), and streptomycin (100 μg/mL) and incubated at 37 °C in a humidified atmosphere containing 5% CO_2_. The cell culture dishes and plates were obtained from Wuxi NEST Biotechnology. Co., Ltd.

### Synthesis and characterization of GNRs

The GNRs were synthesized in an aqueous solution using a seed-mediated growth method^55^. The gold seeds were prepared by mixing 5 mL of 0.5 mM HAuCl_4_ with 5 mL of 0.2 M CTAB, followed by the addition of 600 μL freshly prepared ice-cold 10 mM NaBH_4_ drop-wise under mild stirring. The seed solution was left for 2 h before use. For the synthesis of the GNRs, 18 mL of 5 mM HAuCl_4_ and 225 μL of 0.1 M AgNO_3_ were added to 90 mL of 0.2 M CTAB, and then 200 μL of 1.2 M HCl and 10.5 mL of 10 mM ascorbic acid were added and gently swirled as the color changed from dark orange to colorless. Next, 120 μL of the CTAB-stabilized gold seed solution was rapidly injected. The resulting solution was gently mixed for 10 s and left undisturbed overnight. Finally, the GNR solution was centrifuged at 12,000 rpm for 15 min to stop the reaction. The supernatant was removed, and the precipitate was resuspended in ultrapure water. GNRs of various length-diameter ratios were synthesized by changing the volume of AgNO_3_. The GNR concentration was estimated to be approximately 0.65 nM according to the extinction coefficients at the LSPR wavelength^56^.

The multilayer polyelectrolyte-coated GNRs were synthesized via a layer-by-layer approach^26^,^27^,^57^. A 20 mL volume of 10 mg/mL polyelectrolyte (PAH or PSS) in 1 mM NaCl and 10 mL of 10 mM NaCl were added to 100 mL of 0.65 nM GNRs and stirred for 1 h at room temperature. A centrifugation cycle of 10,000 rpm for 10 min was performed to remove the excess polyelectrolyte and NaCl, and the pellet was re-dispersed in 100 mL of ultrapure water. The second layer of polyelectrolyte was formed using the same method.

Absorption spectra were collected on a TU-1810 UV-Vis-NIR spectrophotometer (Purkinje General Instrument Co. Ltd. Beijing, China). The zeta potentials of the samples were determined using a Zetasizer (Nano ZS90, Malvern Instruments, UK) at 25 °C.

### Cell viability and apoptosis assays

The cell viability was determined by a trypan blue dye exclusion assay according to established protocols, and the cells were counted using a hemocytometer. Apoptosis analysis was evaluated with a sub-G1 assay. The cells were harvested and washed with PBS, followed by fixation with 70% alcohol overnight at 4 °C. The fixed cells were collected, washed with PBS and then stained with 4 μL of 10 mg/mL propidium iodide (PI) and 10 μL of 1 mg/mL RNase. The stained cells were assessed on a flow cytometer (Beckman). The data were processed with FlowJo.

### Western blot analysis

Following the different treatments indicated in the figure legends, the cells were collected and washed with PBS and then lysed with 1% SDS on ice. Then, the cell lysates were heated at 95 °C for 20 minutes and centrifuged at 12,000 *g* for 10 minutes. The supernatant was collected, and the protein concentration was determined with a bicinchoninic acid protein assay kit (Pierce). Samples were run on SDS-PAGE gels and immunoblotted with the antibodies mentioned above.

### Measurement of intracellular ROS level and mitochondrial membrane potential

FACS analysis was carried out to study the intracellular ROS and mitochondrial membrane potential. Briefly, after treatment, the cells were collected, washed with PBS and resuspended in serum-free medium containing the corresponding dye. The intracellular ROS levels were measured by the addition of 10 μM 5-(and-6)-carboxy-2′,7′-dichlorodihydrofluorescein diacetate (carboxy-H_2_DCFDA; Invitrogen) at 37 °C for 20 min. The mitochondrial membrane potential was determined by measuring the retention of the Rh123 dye. The cells were washed again and subjected to flow cytometry analysis. The results were analyzed by FlowJo software.

### Fluorescence microscopy of LC3 and acridine orange staining

HCT116 cells were preliminarily cultured in 12-well plates overnight on glass slides. To inspect autophagic fluorescent puncta accumulation in LC3, the cells were transiently transfected with the pEGFP-LC3 plasmid using FuGENE^TM^ HD (Roche) according to the manufacturer’s protocol. After treatment, the cells were observed with a fluorescent microscope (Olympus BX51).

Autophagy is the process of sequestering cytoplasmic proteins into the lytic component and is characterized by the formation and promotion of acidic vesicular organelles (AVOs), as described previously. To detect the development of AVOs, the cells treated on glass slides were stained with acridine orange (1 μg/mL) for 15 min and then observed with a fluorescent microscope (Olympus BX51).

### Transmission electron microscopy (TEM)

After different treatments as indicated, the cells were collected and washed with PBS. The cells were then soaked in 2.5% glutaraldehyde for fixation, washed three times in 0.1 M PBS and fixed with 1% OsO_4_. Next, the cells were dehydrated using a range of alcohol concentrations for 15 minutes. The cells were then embedded into paraffin and sliced using an LKB-V ultramicrotome (BROMMA, Sweden). For TEM inspection, the prepared sections were examined under an H-600 transmission electron microscope (Hitachi, Japan).

### Animal experiments and inductively coupled plasma mass spectrometry (ICP-MS)

Male 6-week-old BALB/c mice were obtained from the Disease Prevention Center of Hubei Province (Wuhan, Hubei, China). In total, 8 mice were used to study the biodistribution of GNRs at two time points, with four mice per time point. Mice were inoculated with the C6 cells. Approximately 2 × 10^6^ cells/100 μL were subcutaneously injected into the right thighs of the mice. When the tumors attained a size of 8–10 mm in diameter, we performed intravenous delivery of 200 μl of 0.9% saline containing GNRs at a dose of 560 μg/kg weight. The mice were anesthetized with ether, weighed, and killed at 4 h and 24 h. The heart, liver, spleen, lungs, kidneys, and tumor tissues were collected and washed three times with deionized water to remove residual blood. After the extra water on the surface had been removed with filter paper, each tissue was weighed for quantitative analysis of the GNRs in tissues.

For quantitative measurement, all tissues were weighed, digested, and measured by ICP-MS. Briefly, 3 mL HNO_3_ was added to a conical flask to predigest the samples overnight. The samples were mixed with 2 mL H_2_O_2_ and were digested and heated for approximately 3 h at 150 °C in open vessels on a hot plate. The remaining solution was cooled and diluted to 3 mL with a mixed acid solution containing 2% HNO_3_ and 1% HCl. In the process of quantitative analysis, a blank solution and a series of gold standard solutions (0.1, 0.5, 1, 5, 10, 50, 100, and 200 ppb) were prepared with the mixture solution and were measured to obtain the detection limit and a standard curve. Bismuth (10 ppb) in a mixed acid solution was used as an internal standard solution. Both the standard and the test solutions were measured three times by ICP-MS, whereas the blank solution was measured additional times.

### Malondialdehyde (MDA) assay and serum ALT activity assay

Twelve male 6-week-old BALB/c mice were used to study the toxicity of the GNRs, including the MDA level and ALT activity. The mice were injected intravenously with 200 μL of a 0.9% saline solution containing GNRs at a dose of 560 μg/kg weight every other day for two weeks. The liver tissues and blood samples from the mice were harvested after all the mice were sacrificed.

The liver tissue samples were homogenized and sonicated in RIPA buffer on ice. Tissue lysates were then centrifuged at 12,000 g for 15 min at 4 °C to collect the supernatant. The liver tissues and blood tissues from the mice were subjected to an MDA assay as described in the lipid peroxidation MDA assay kit (Beyotime, Nantong, China). The MDA concentration of each sample was detected using a multimode microplate reader (SpectramMax M5) at 532 nm, using 490 nm as a control. The blood samples from the mice were also subjected to serum ALT activity analysis as described in the serum ALT assay kit (Nanjing Jiancheng Bioengineering Institute, Nanjing, China). The ALT levels were detected using a multimode microplate reader (SpectraMax M5) at 510 nm.

### Statistical analysis

All experiments were performed at least three times. Student’s t-test was used for all the statistical analyses, and the differences were considered significant if the p value was less than 0.05.

## Additional Information

**How to cite this article**: Wan, J. *et al.* Surface chemistry but not aspect ratio mediates the biological toxicity of gold nanorods *in vitro* and *in vivo*. *Sci. Rep.* **5**, 11398; doi: 10.1038/srep11398 (2015).

## Supplementary Material

Supplementary Information
