# Supplementary material for: Surface chemistry but not aspect ratio mediates the biological toxicity of gold nanorods in vitro and in vivo
Source: Sci Rep. 2015 Jun 22;5:11398. doi: 10.1038/srep11398 (PMC4476041; doi:10.1038/srep11398)
Supplement: Supplementary Information [file srep11398-s1.pdf]

Surface chemistry but not aspect ratio mediates the biological toxicity of gold nanorods in vitro and in vivo

Jiali Wan[1], Jia-Hong Wang[2], Ting Liu[1], Zhixiong Xie[1], Xue-Feng Yu[2], Wenhua Li[1*]

[1]College of Life Sciences, Wuhan University, Wuhan 430072, P R China;
[2]Key Laboratory of Artificial Micro- and Nano-Structures of Ministry of Education, School of Physics and Technology, Wuhan University, Wuhan 430072, China

*Corresponding author: Wenhua Li
Email: whli@whu.edu.cn
Tel: +86-027-68756711
Fax: +86-027-68756711

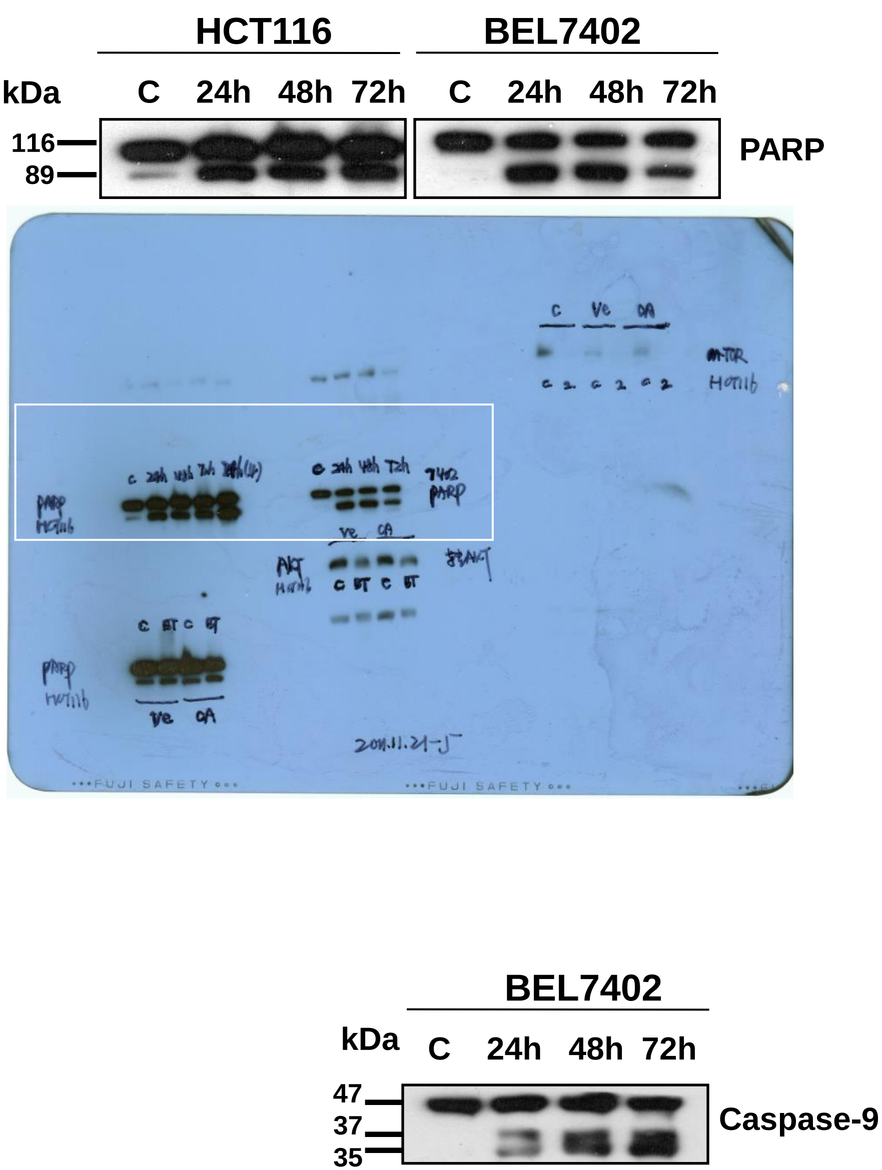

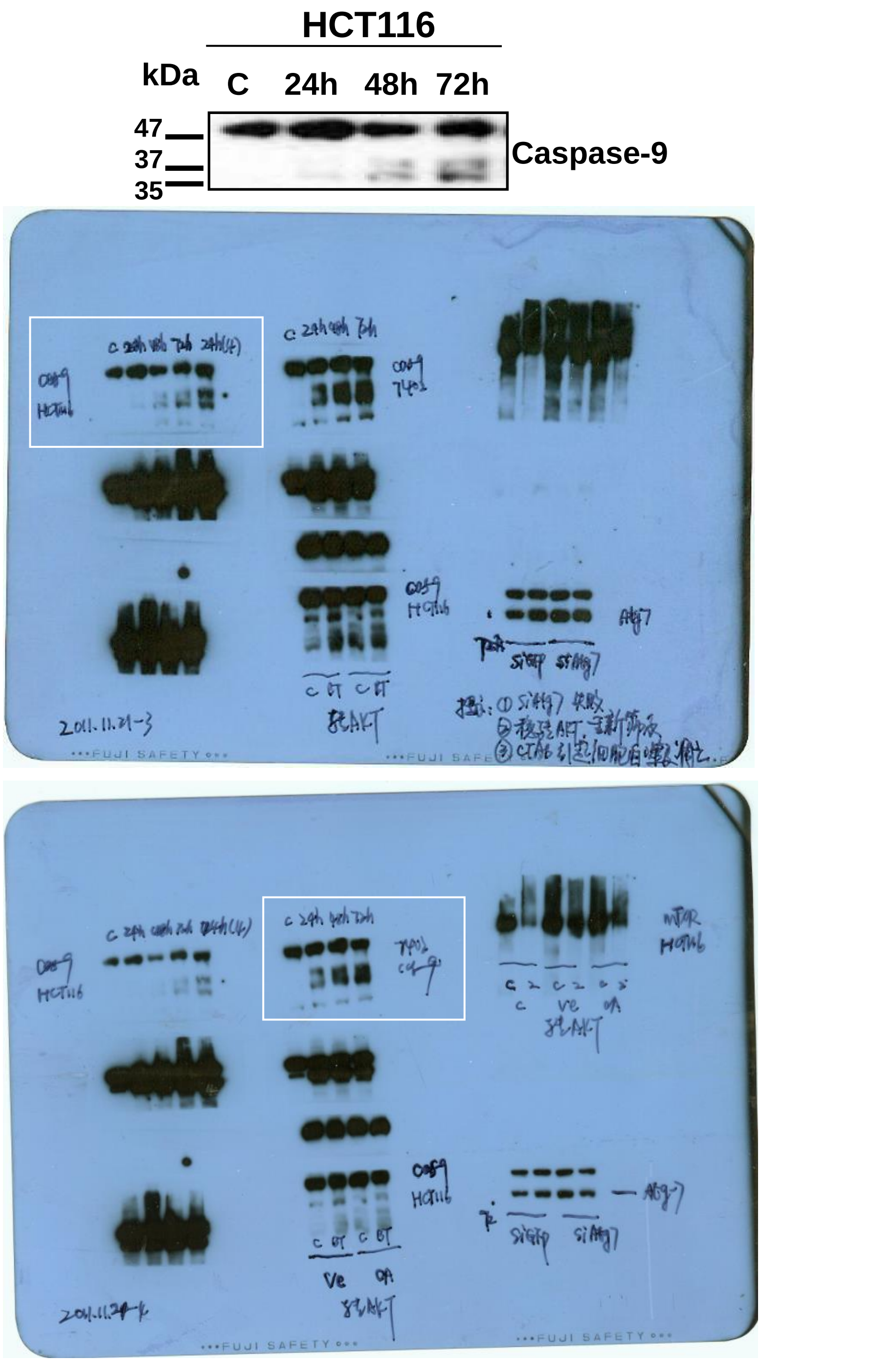

Supplementary Figure 1

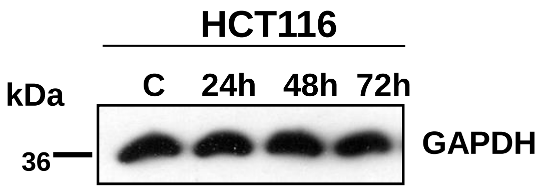

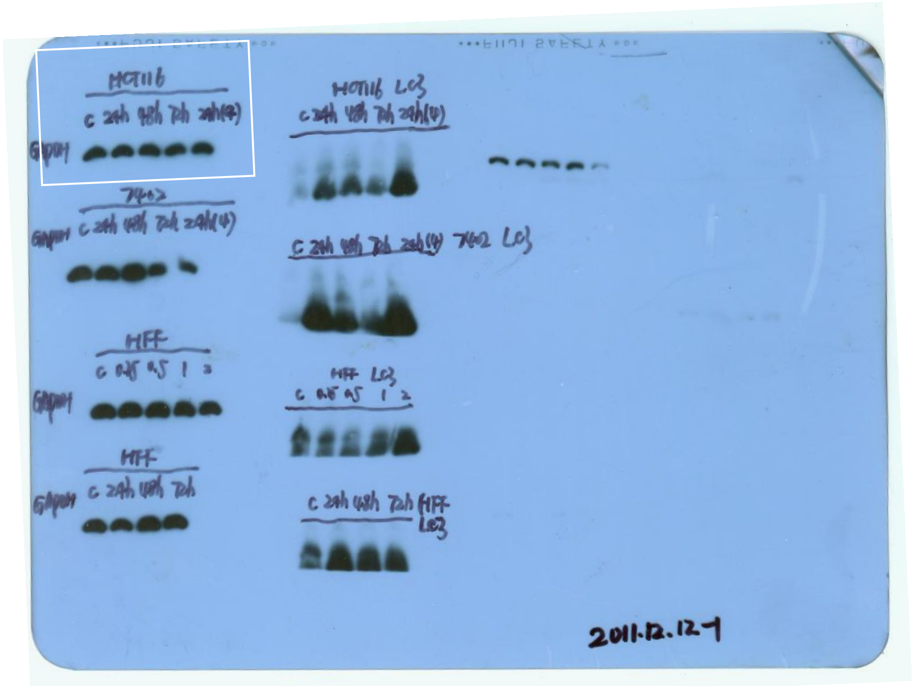

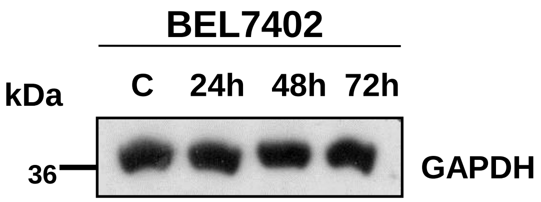

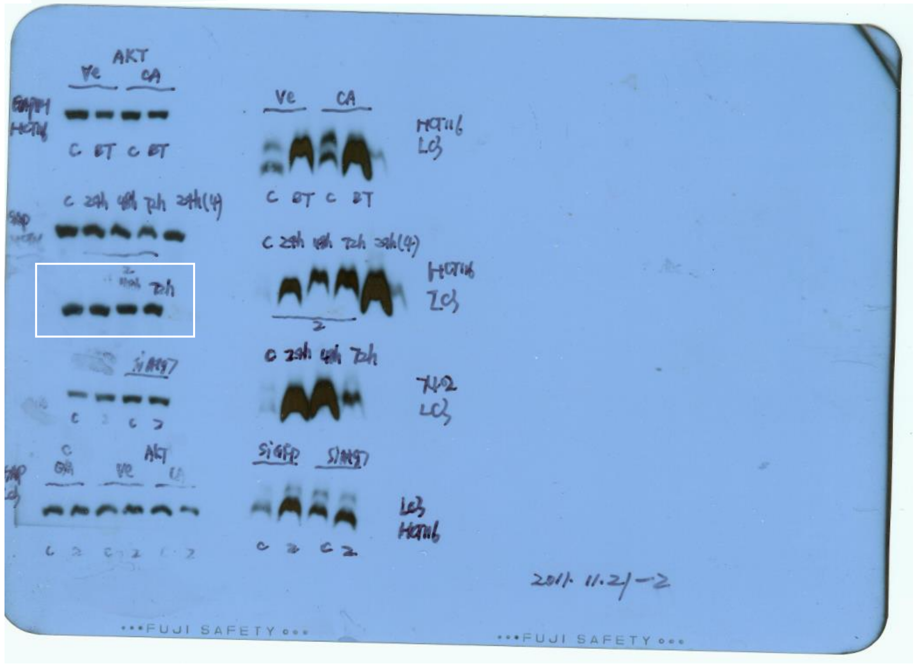

Supplementary Figure 1

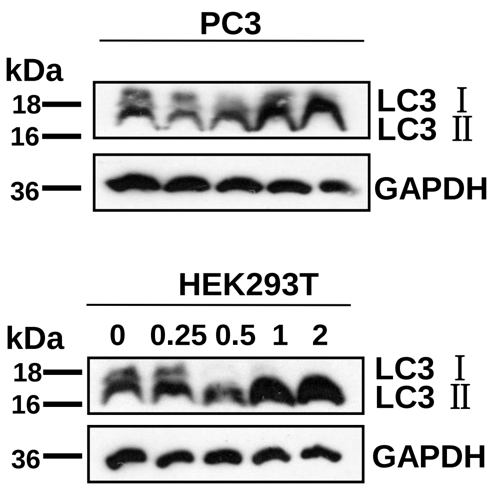

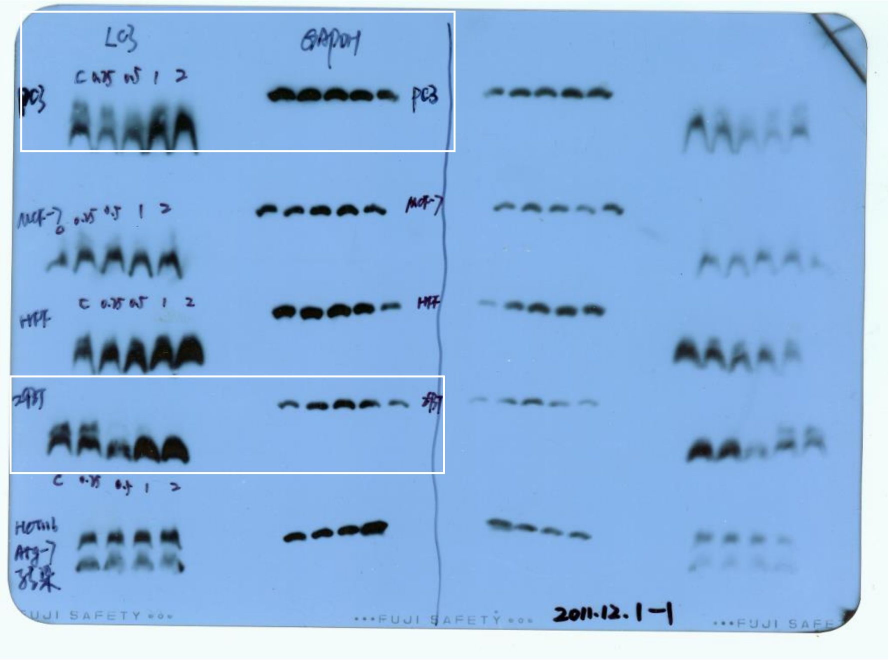

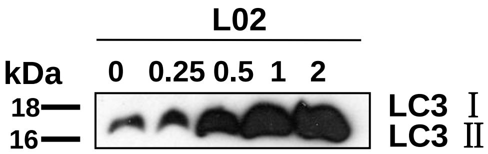

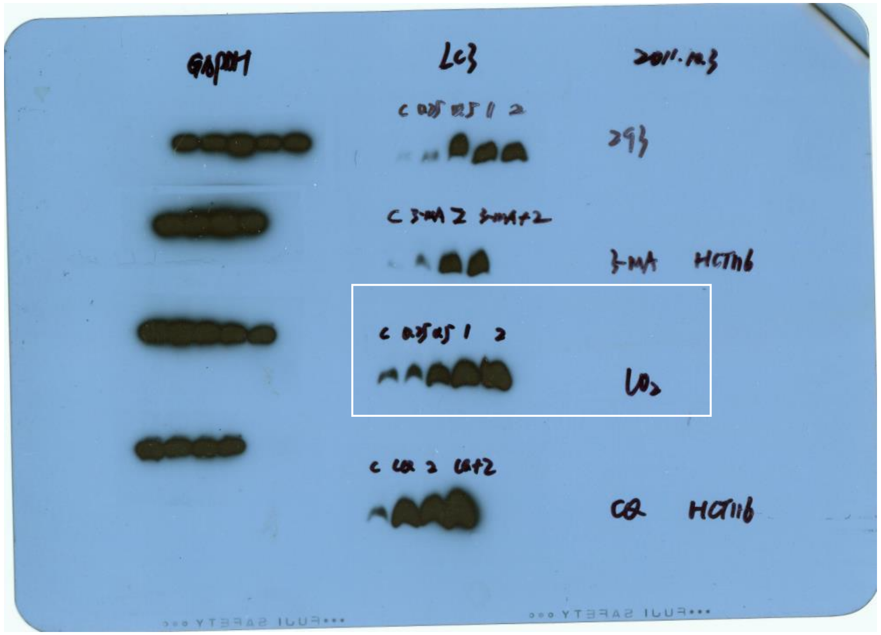

Supplementary Figure 2

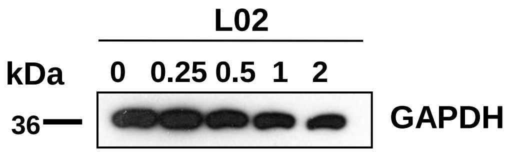

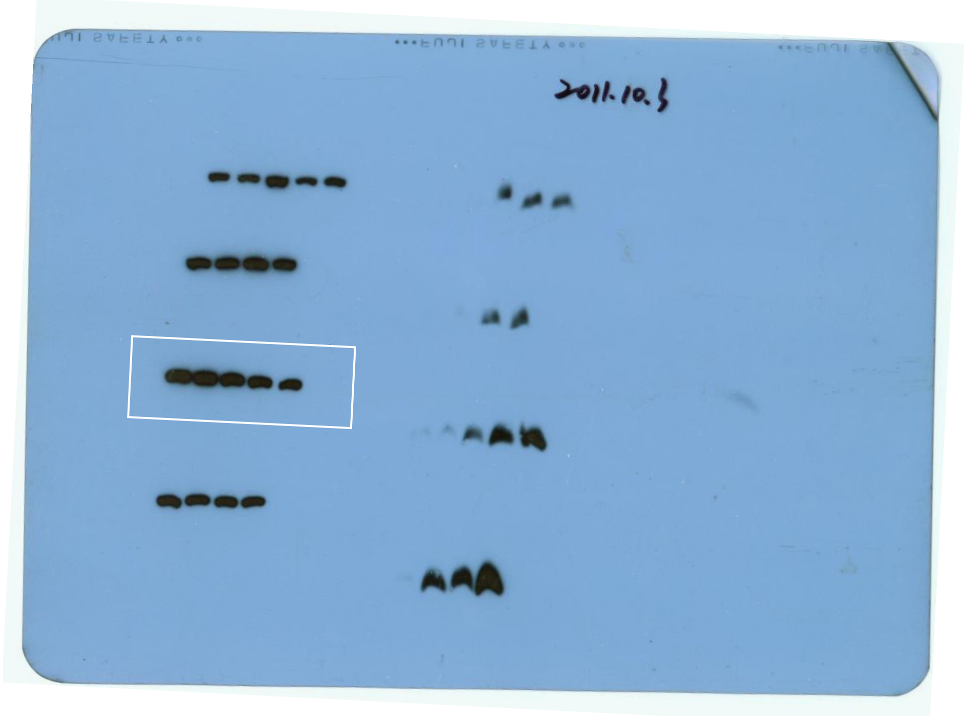

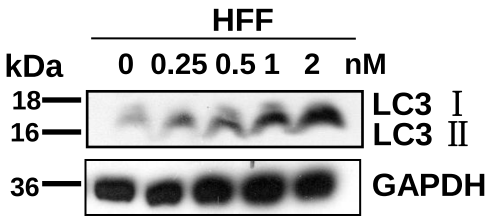

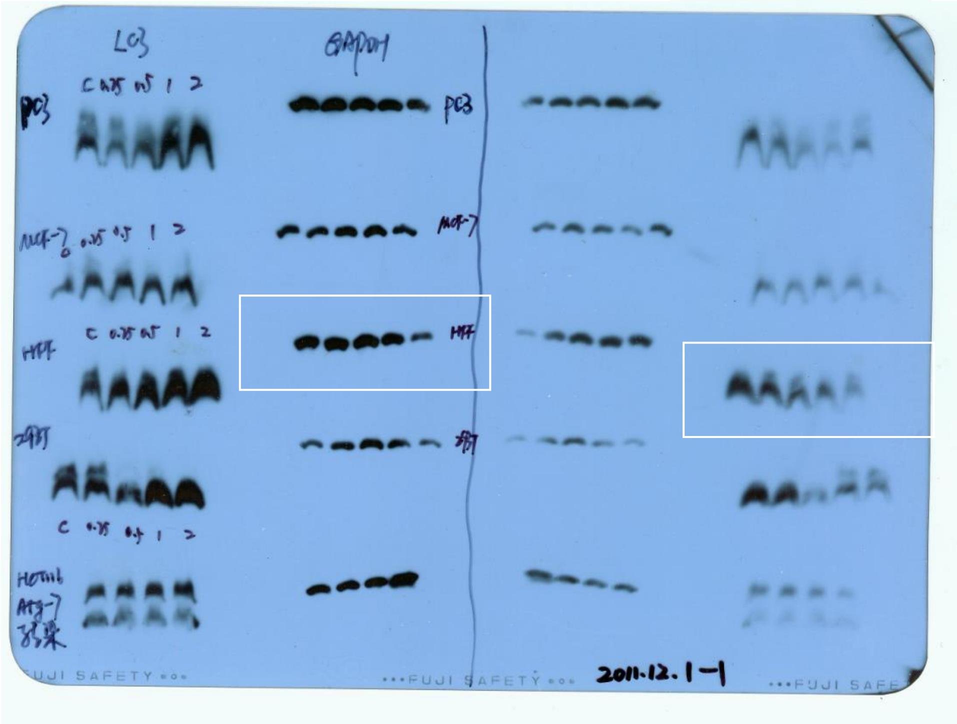

Supplementary Figure 2

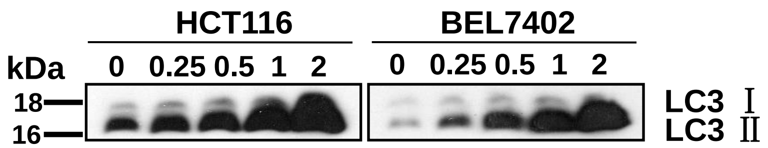

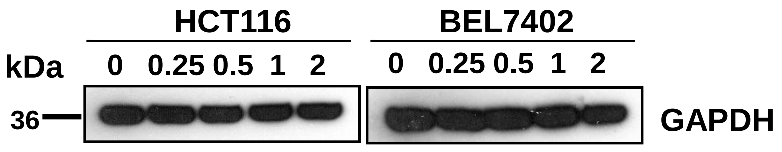

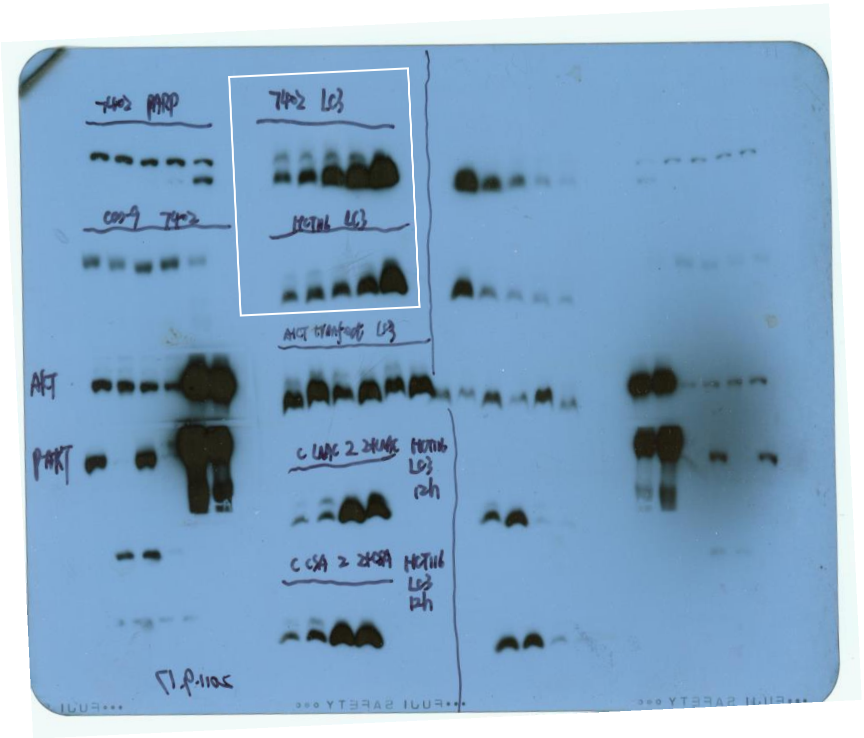

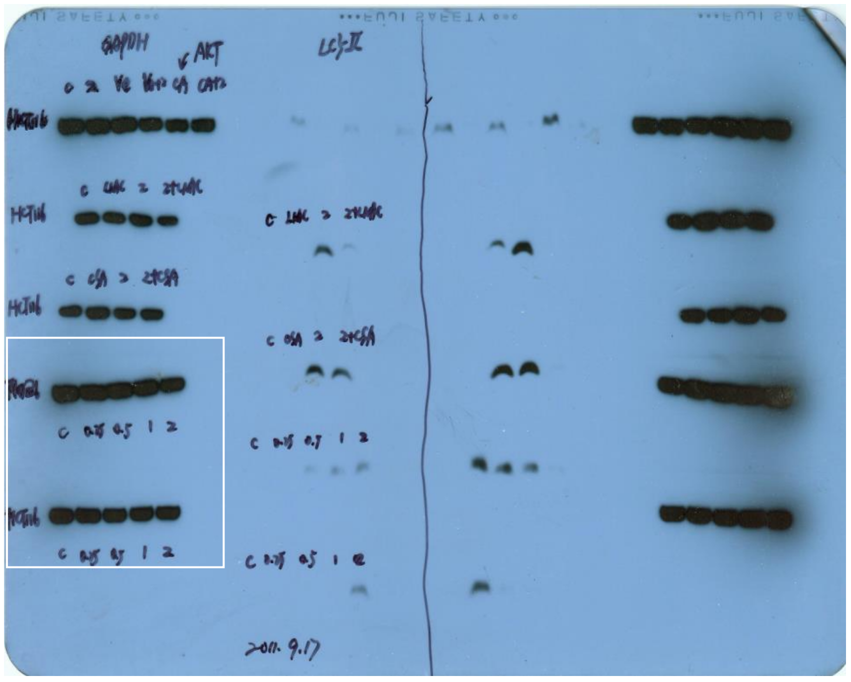

Supplementary Figure 2

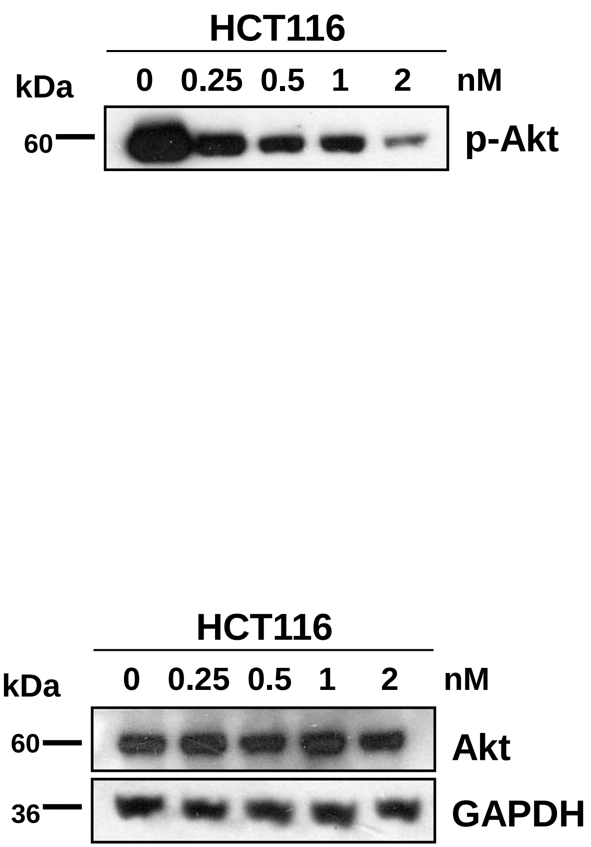

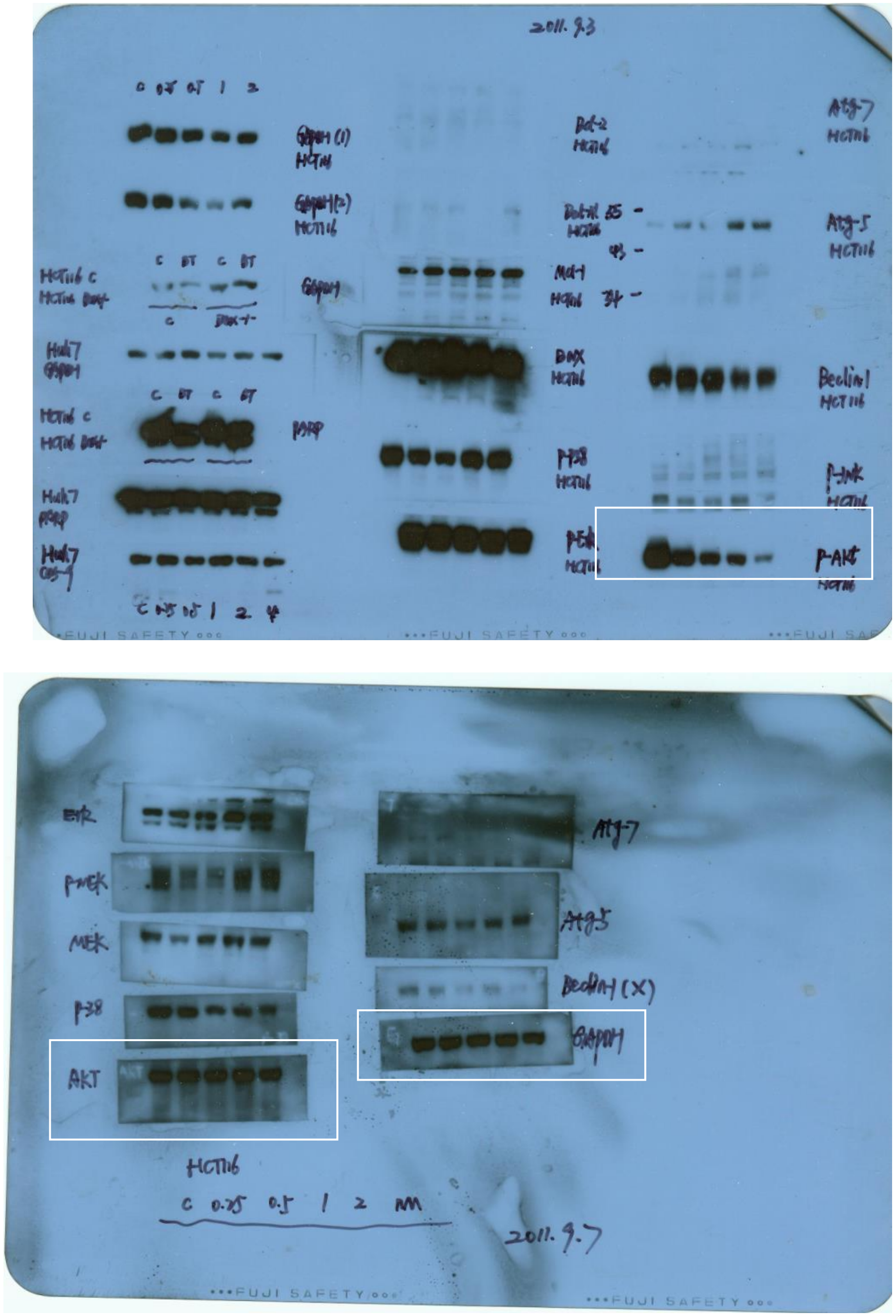

Supplementary Figure 2

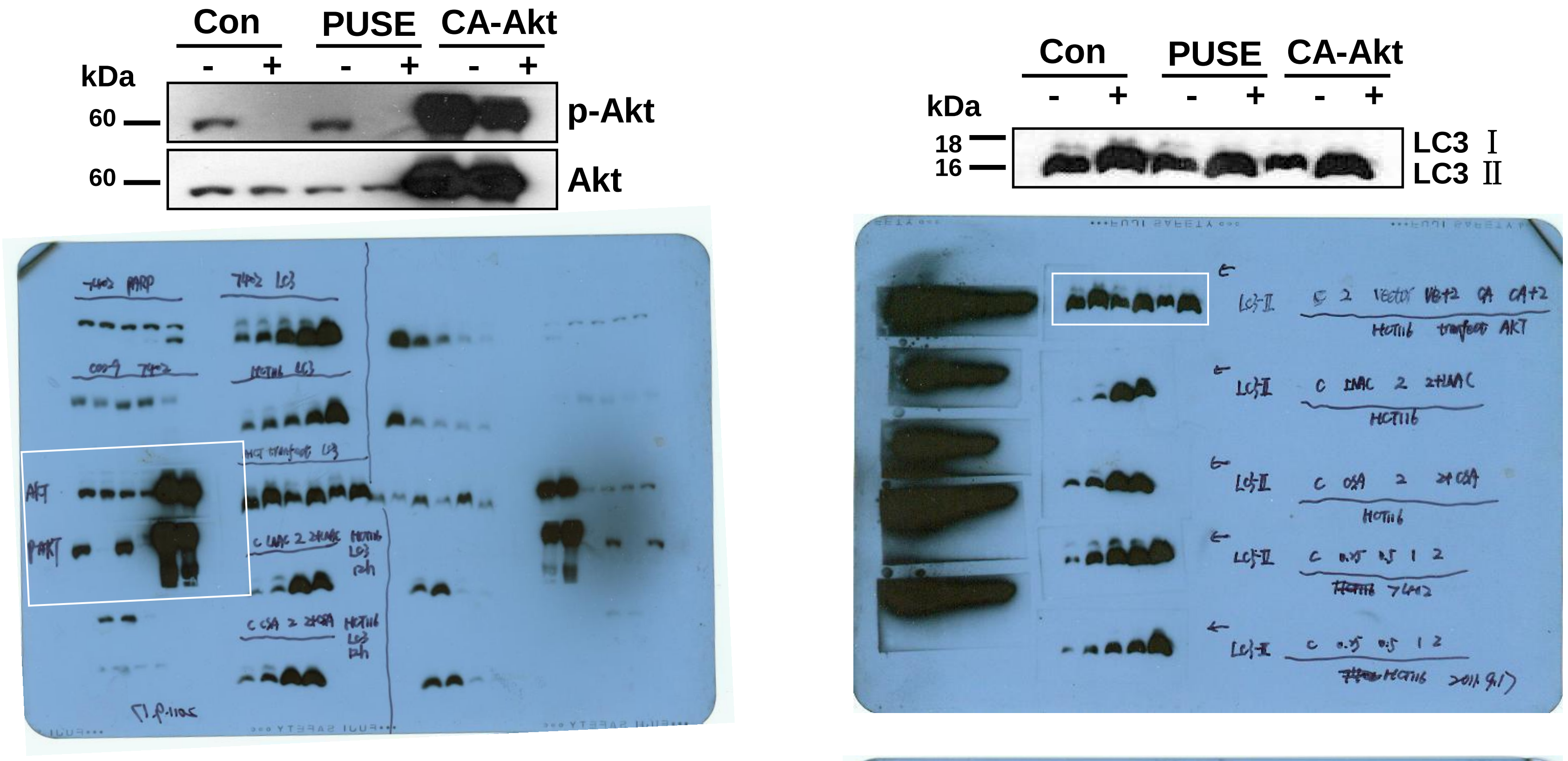

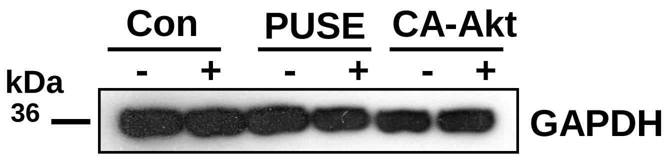

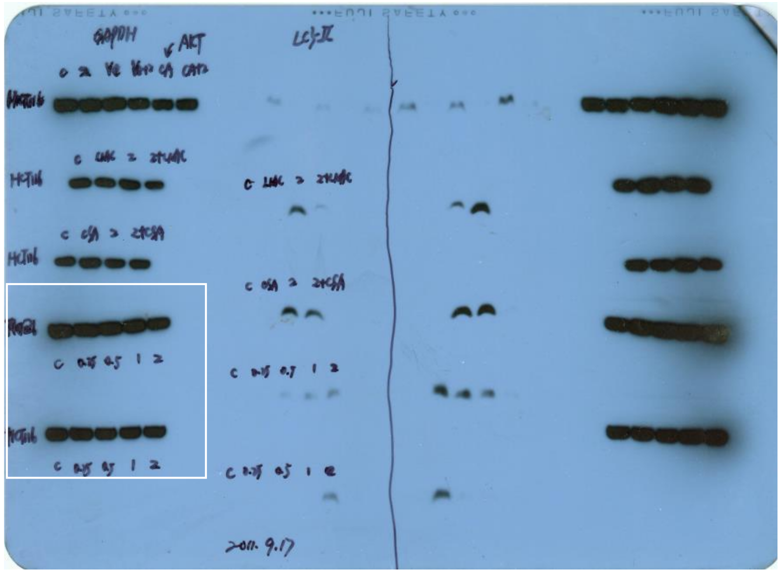

Supplementary Figure 2

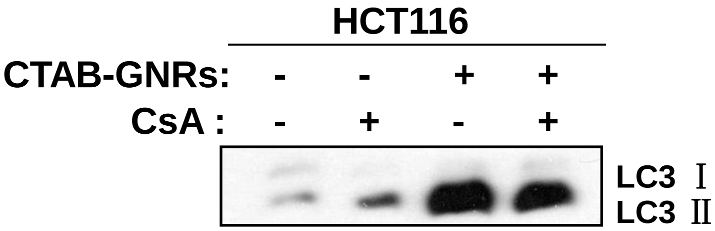

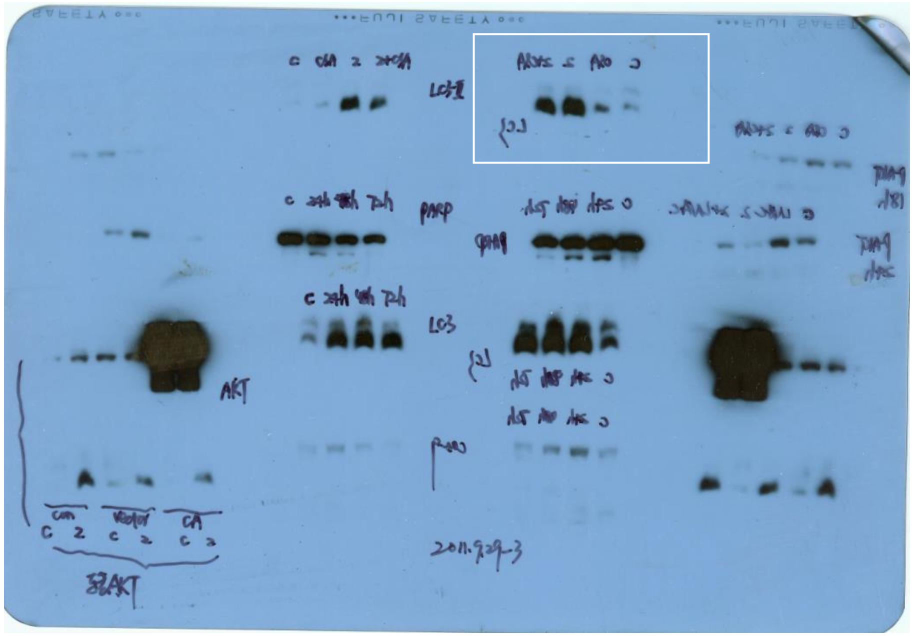

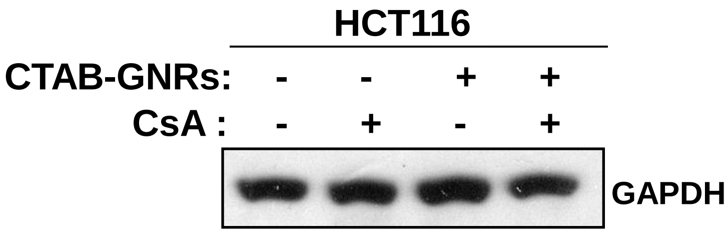

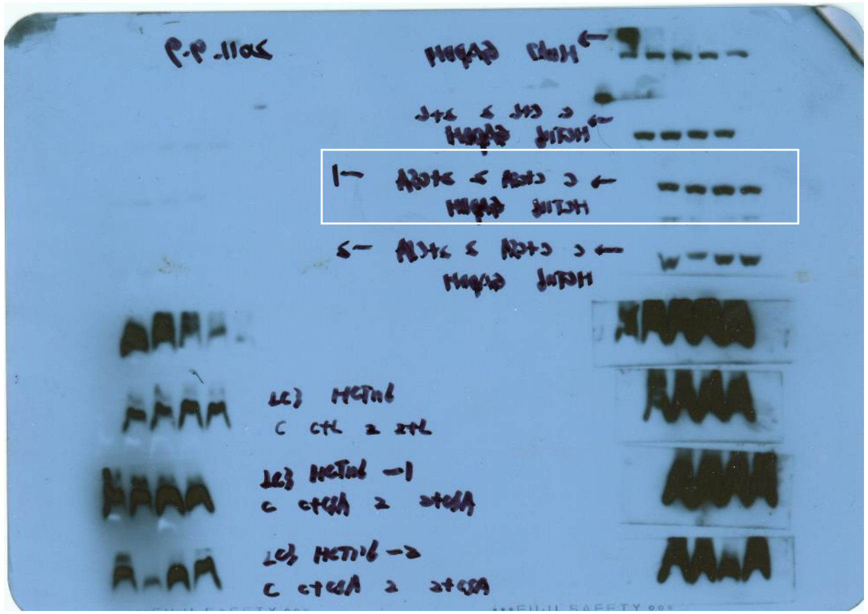

Supplementary Figure 3

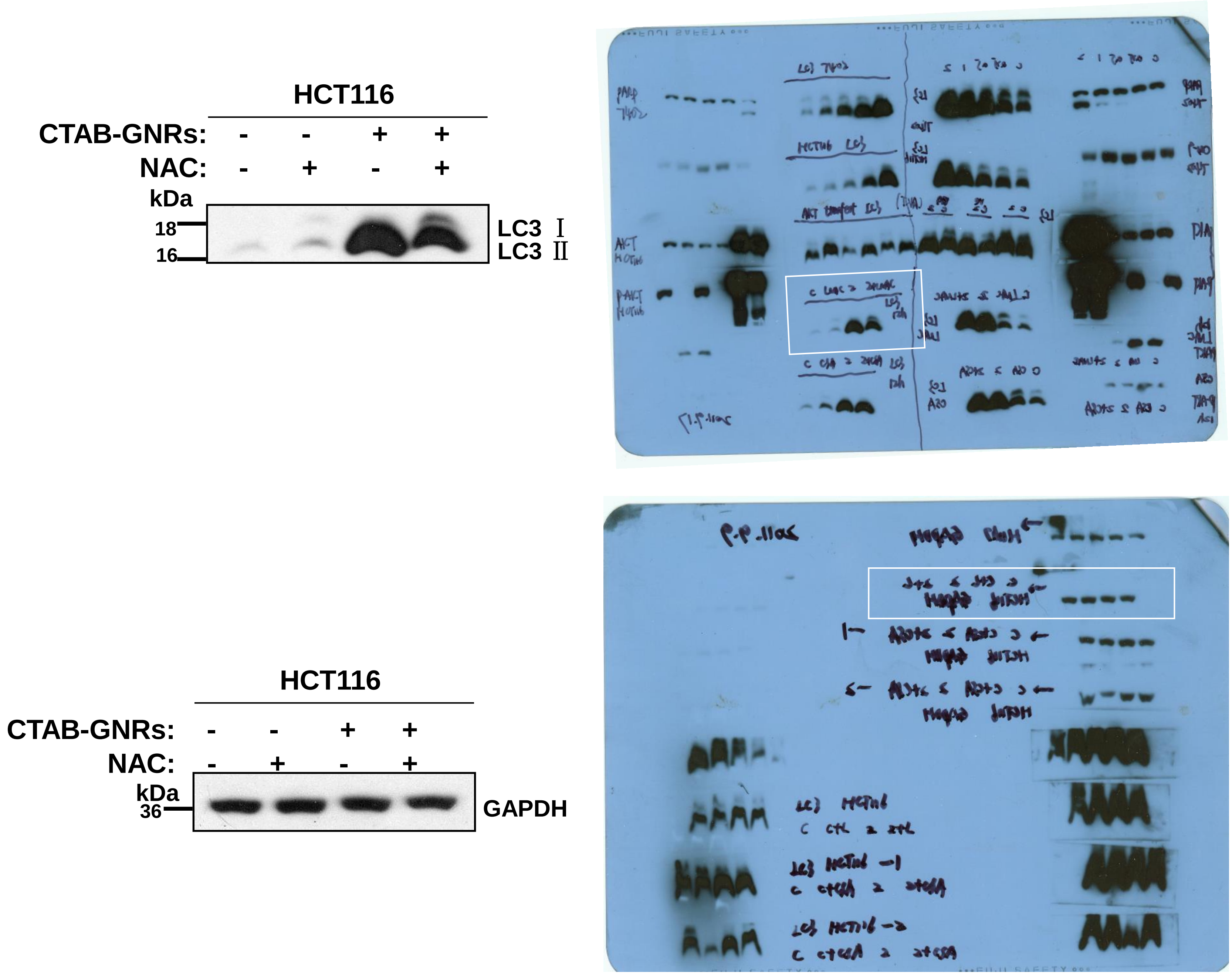

Supplementary Figure 4

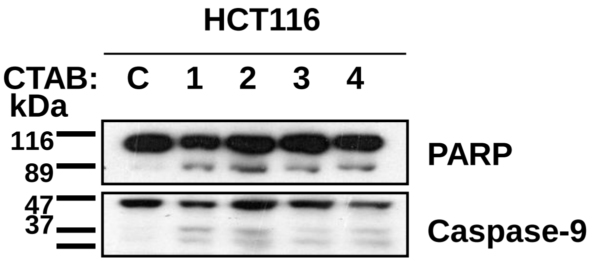

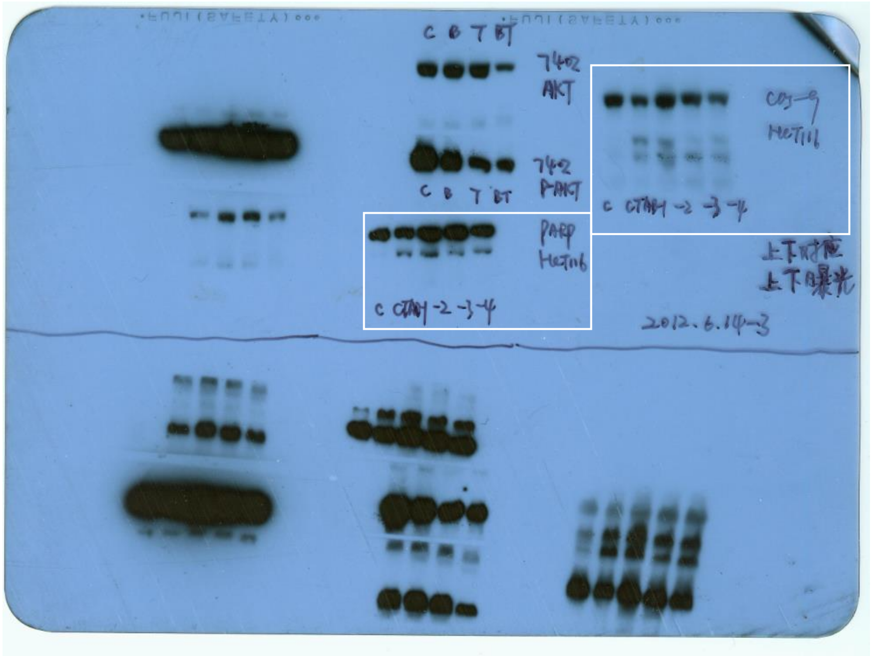

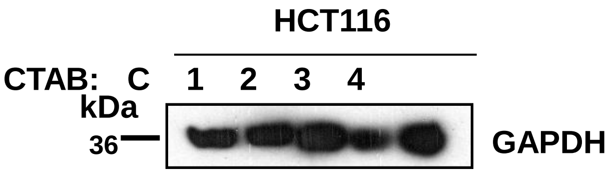

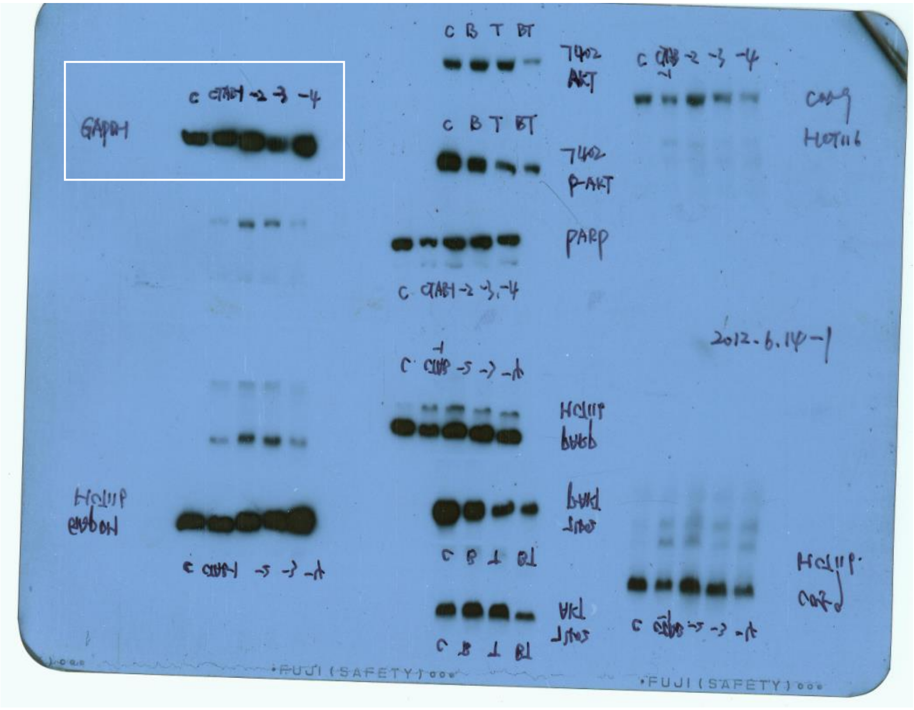

Supplementary Figure 5

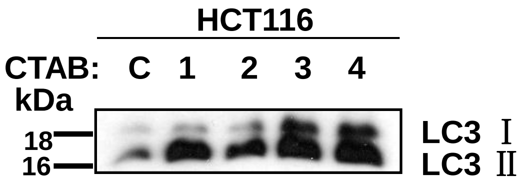

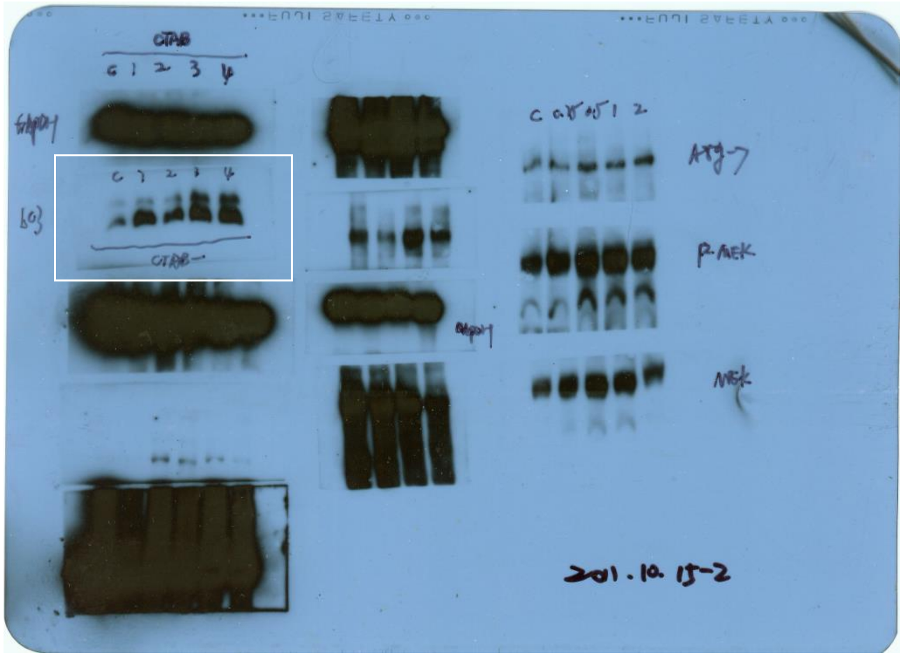

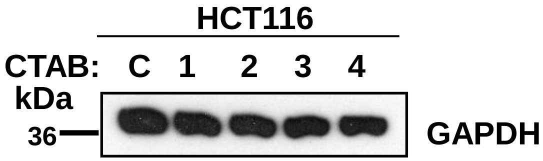

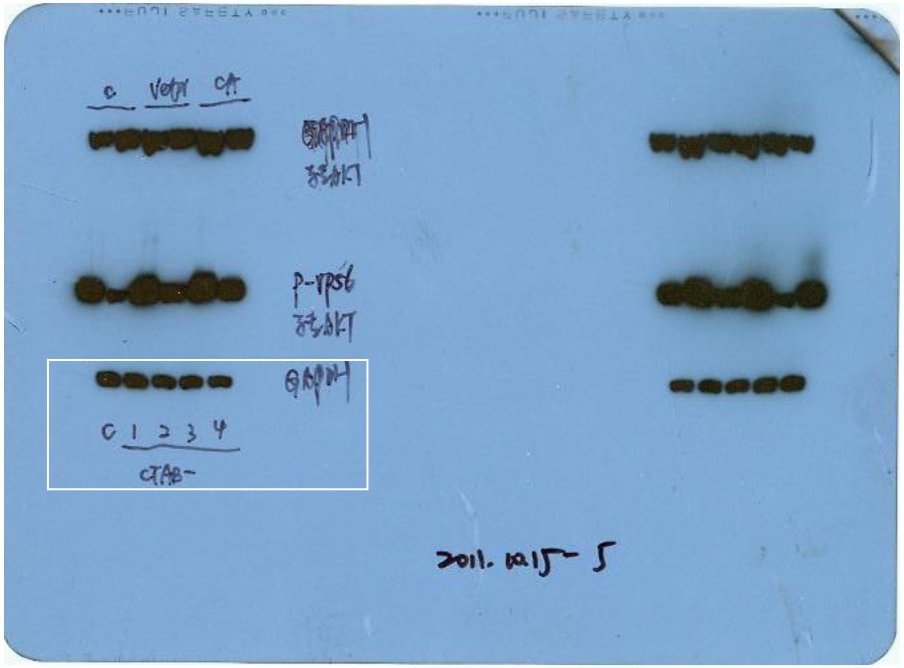

Supplementary Figure 5

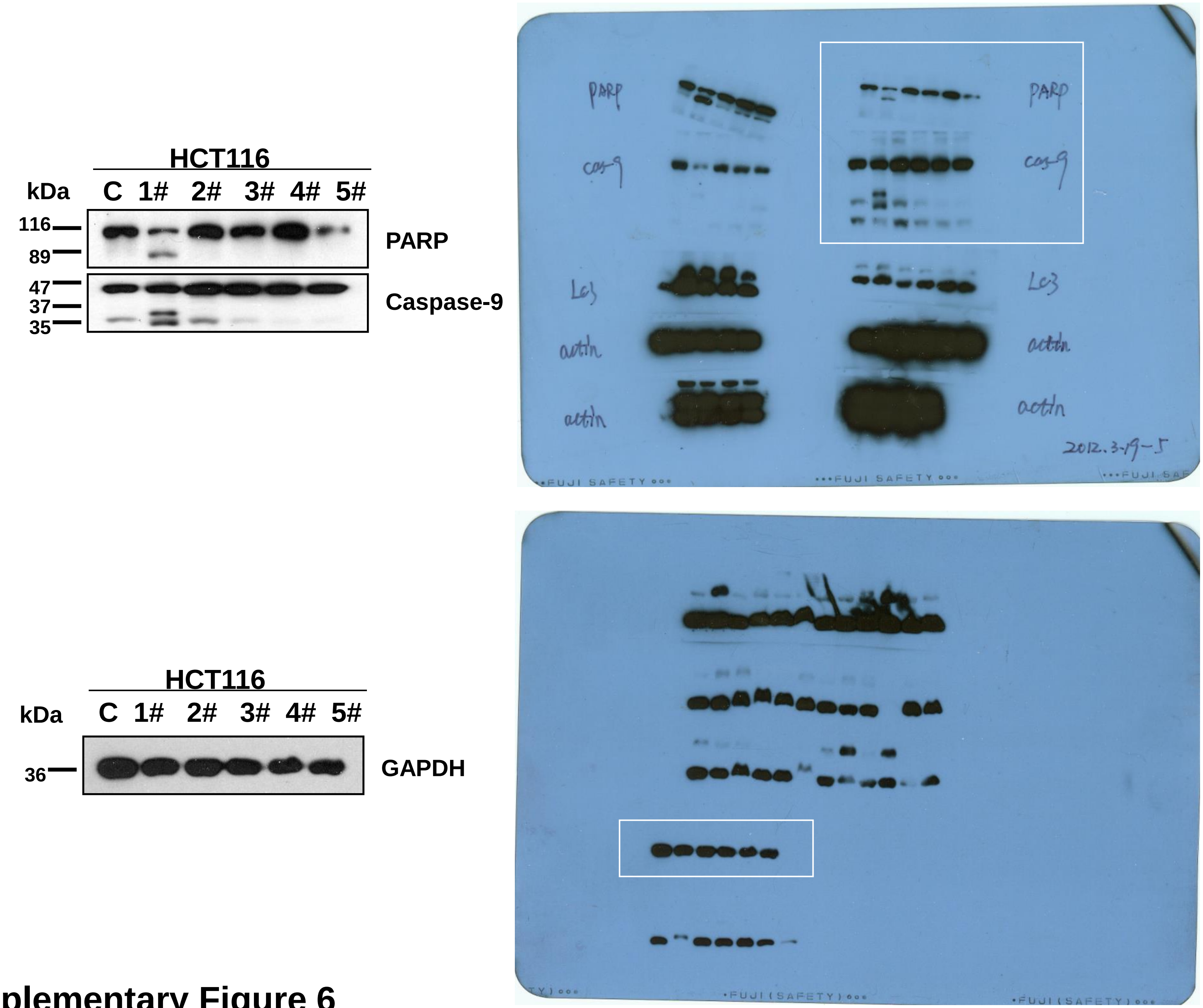

Supplementary Figure 6

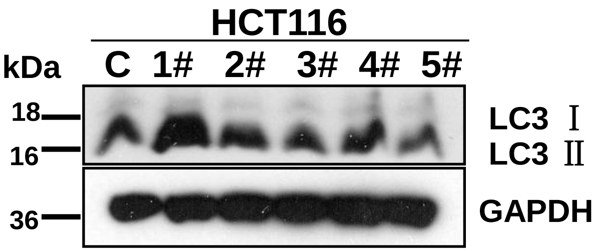

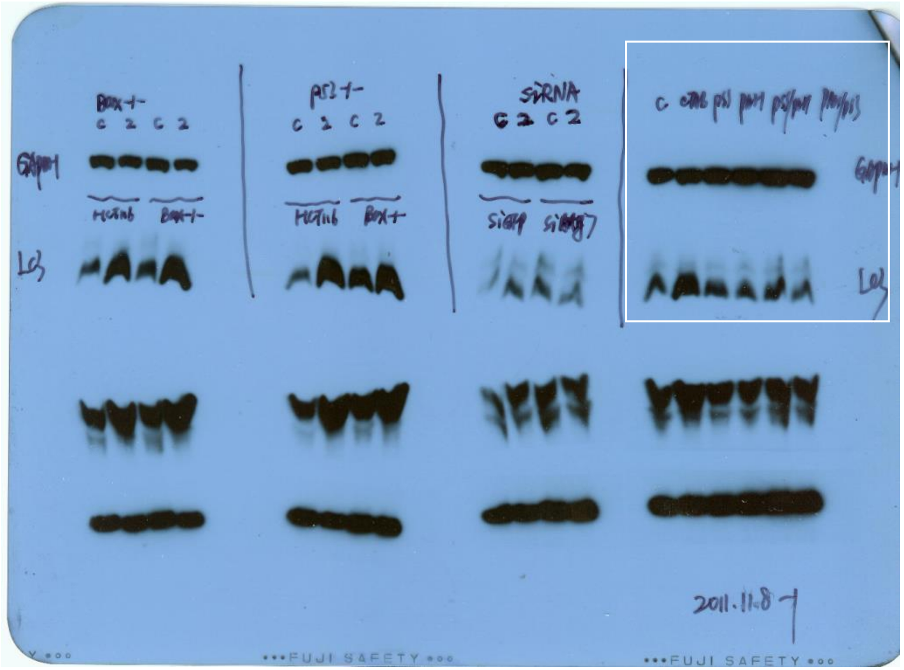

Supplementary Figure 6
